# Effects of a mindfulness-based versus a health self-management intervention on objective cognitive performance in older adults with subjective cognitive decline (SCD): a secondary analysis of the SCD-Well randomized controlled trial

**DOI:** 10.1186/s13195-022-01057-w

**Published:** 2022-09-06

**Authors:** Tim Whitfield, Harriet Demnitz-King, Marco Schlosser, Thorsten Barnhofer, Eric Frison, Nina Coll-Padros, Sophie Dautricourt, Florence Requier, Marion Delarue, Julie Gonneaud, Olga M. Klimecki, Antoine Lutz, Léo Paly, Eric Salmon, Ann-Katrin Schild, Zuzana Walker, Frank Jessen, Gaël Chételat, Fabienne Collette, Miranka Wirth, Natalie L. Marchant, The Medit-Ageing Research Group, Amélie Michon, Raquel Sanchez-Valle, Claudia Schwars, Cindy Lai, Roxane Coueron, Eider M. Arenaza-Urquijo, Géraldine Poisnel, Floriane Delphin-Combe, Julien Asselineau, Pierre Krolak-Salmon, José Luis Molinuevo, Florence Allais, Romain Bachelet, Viviane Belleoud, Clara Benson, Beatriz Bosch, Maria Pilar Casanova, Hélène Espérou, Karine Goldet, Idir Hamdidouche, Maria Leon, Dix Meiberth, Hendrik Mueller, Theresa Mueller, Valentin Ourry, Leslie Reyrolle, Ana Salinero, Lena Sannemann, Yamna Satgunasingam, Hilde Steinhauser, Patrik Vuilleumier, Cédrick Wallet, Janet Wingrove

**Affiliations:** 1 Division of Psychiatry, University College London, 6th Floor Maple House, 149 Tottenham Court Road, London, W1T 7NF UK; 2 Department of Psychology, Faculty of Psychology and Educational Sciences, University of Geneva, Geneva, Switzerland; 3 School of Psychology, University of Surrey, Guildford, UK; 4 Bordeaux Population Health Center, University of Bordeaux, INSERM, EUCLID/F-CRIN Clinical Trials Platform, CHU Bordeaux, F-33000 Bordeaux, France; 5 Service d’information médicale, CHU Bordeaux, Bordeaux, France; 6 Alzheimer’s Disease and Other Cognitive Disorders Unit, Hospital Clinic, IDIBAPS, Barcelona, Spain; 7 Normandie University, UNICAEN, INSERM, U1237, PhIND “Physiopathology and Imaging of Neurological Disorders”, Institut Blood and Brain @ Caen-Normandie, Cyceron, 14000 Caen, France; 8 Neurology Department, University Hospital, Caen, France; 9 GIGA-CRC In Vivo Imaging, University of Liège, Liège, Belgium; 10 Psychology and Neuroscience of Cognition Research Unit, University of Liège, Liège, Belgium; 11 Clinical Psychology and Behavioral Neuroscience, Faculty of Psychology, Technische Universität Dresden, Dresden, Germany; 12 Lyon Neuroscience Research Center Inserm U1028, CNRS UMR5292, Lyon 1 University, Lyon, France; 13 Department of Psychiatry, Medical Faculty, University of Cologne, Cologne, Germany; 14 Essex Partnership University NHS Foundation Trust, Wickford, UK; 15 Excellence Cluster on Cellular Stress Responses in Aging-Associated Diseases (CECAD), University of Cologne, Cologne, Germany; 16 German Center for Neurodegenerative Diseases (DZNE), Bonn, Germany; 17 German Center for Neurodegenerative Diseases (DZNE), Dresden, Germany

**Keywords:** Mindfulness, Compassion, Cognition, Subjective cognitive decline, Randomized controlled trial

## Abstract

**Background:**

Older individuals with subjective cognitive decline (SCD) perceive that their cognition has declined but do not show objective impairment on neuropsychological tests. Individuals with SCD are at elevated risk of objective cognitive decline and incident dementia. Non-pharmacological interventions (including mindfulness-based and health self-management approaches) are a potential strategy to maintain or improve cognition in SCD, which may ultimately reduce dementia risk.

**Methods:**

This study utilized data from the SCD-Well randomized controlled trial. One hundred forty-seven older adults with SCD (*M*_Age_ = 72.7 years; 64% female) were recruited from memory clinics in four European countries and randomized to one of two group-based, 8-week interventions: a Caring Mindfulness-based Approach for Seniors (CMBAS) or a health self-management program (HSMP). Participants were assessed at baseline, post-intervention (week 8), and at 6-month follow-up (week 24) using a range of cognitive tests. From these tests, three composites were derived—an “abridged” Preclinical Alzheimer’s Cognitive Composite 5 (PACC5_Abridged_), an attention composite, and an executive function composite. Both per-protocol and intention-to-treat analyses were performed. Linear mixed models evaluated the change in outcomes between and within arms and adjusted for covariates and cognitive retest effects. Sensitivity models repeated the per-protocol analyses for participants who attended ≥ 4 intervention sessions.

**Results:**

Across all cognitive composites, there were no significant time-by-trial arm interactions and no measurable cognitive retest effects; sensitivity analyses supported these results. Improvements, however, were observed within both trial arms on the PACC5_Abridged_ from baseline to follow-up (Δ [95% confidence interval]: CMBAS = 0.34 [0.19, 0.48]; HSMP = 0.30 [0.15, 0.44]). There was weaker evidence of an improvement in attention but no effects on executive function.

**Conclusions:**

Two non-pharmacological interventions conferred small, non-differing improvements to a global cognitive composite sensitive to amyloid-beta-related decline. There was weaker evidence of an effect on attention, and no evidence of an effect on executive function. Importantly, observed improvements were maintained beyond the end of the interventions. Improving cognition is an important step toward dementia prevention, and future research is needed to delineate the mechanisms of action of these interventions and to utilize clinical endpoints (i.e., progression to mild cognitive impairment or dementia).

**Trial registration:**

ClinicalTrials.gov, NCT03005652.

**Supplementary Information:**

The online version contains supplementary material available at 10.1186/s13195-022-01057-w.

## Background

Individuals with subjective cognitive decline (SCD) perceive that their cognition has worsened but do not show impairment on standardized cognitive tests used to detect mild cognitive impairment (MCI) and dementia [1]. It is increasingly recognized that SCD is an etiologically heterogeneous entity, with correspondingly varied clinical outcomes [2, 3]. While most older adults with SCD do not decline to dementia in the near term [4], they are at twice the risk of progression to dementia versus those without SCD [5]. At a group level, memory clinic patients with SCD exhibit modest neuropsychological deficits compared to healthy older adults without SCD [6], and worse cognition predicts progression to dementia in SCD cohorts [4]. Furthermore, SCD is associated with elevated depressive and anxiety symptoms [7], and a recent meta-analysis of longitudinal studies found that the presence of anxiety (but not depressive) symptoms increased the risk of incident MCI and dementia in individuals with SCD by 40% [8].

In response to this accumulating evidence, an increasing number of randomized controlled trials (RCTs) have targeted cognitive and affective outcomes in people with SCD, with the ultimate aim of attenuating dementia risk. However, two systematic reviews concluded that existing RCTs in SCD were of variable quality and that the evidence of efficacy across targeted outcomes was limited [9, 10]. Both syntheses offered numerous recommendations to improve the methodological rigor of the field moving forward; these included encouraging future investigators to characterize participants with SCD more systematically (e.g., according to published criteria), recruit sufficient participants to achieve greater statistical power, define the mechanisms underpinning the hypothesized effects of interventions, include active (rather than inactive) comparators, and measure outcomes at follow-up to evaluate the maintenance of any observed effects.

From a theoretical perspective, mindfulness-based interventions (MBIs) appear a promising approach for ameliorating the cognitive and affective features of SCD. The core components of MBIs are three taught practices (i.e., the body scan, mindful movement, and sitting meditation), conceptualized as means of promoting attentional and emotional self-regulation [11]. By virtue of this dual focus on cognition and affect, MBIs appear well-matched to the clinical profile of SCD. Two recent reviews concluded that MBIs reduce depressive symptoms in older adults, although the evidence for anxiety was mixed [12, 13]. Furthermore, a recent meta-analysis found that MBIs outperformed comparators for objective cognitive function outcomes in older (but not younger) individuals [14]. Health self-management programs (HSMPs) are a commonly used active comparator in MBI RCTs [15–17], although in other studies, they are the primary focus. For example, an RCT involving older women found that a healthy aging psychoeducation group did not outperform a waitlist group on an executive function composite [18]. Another trial evaluating an 8-week health education program in older adults found attention scores were improved versus a waitlist at the post-intervention and 6-month follow-up visits [19].

Here, we report the results of a multinational RCT of a novel MBI versus an HSMP in individuals with SCD, focusing on objective cognitive function outcomes. Given that limited existing work has been conducted in this area, our hypotheses were based on prior meta-analyses which evaluated MBIs in a range of populations, including healthy older adults and individuals with MCI [14, 20]. While these evidence syntheses were not SCD-specific, SCD overlaps with both healthy cognitive aging (both lack objective cognitive impairment) and also MCI (both are associated with increased dementia risk). Thus, following the prior findings that MBIs outperformed comparators in a combined analysis of various cognitive domains [14], we hypothesized that the current MBI would confer greater gains (versus the HSMP) to a global cognitive composite. Given the meta-analysis suggested that the “overall” result was driven by improved executive function [14], we also predicted that the current MBI would confer greater benefits to executive function versus the HSMP. Lastly, two previous meta-analyses found that MBIs did not outperform comparators for attention outcomes in older persons [14, 20]; we thus hypothesized that any improvement in this cognitive domain would not significantly differ between arms in the current trial.

## Methods

### Design

SCD-Well was a European multicenter, observer-blind RCT with two intervention arms: an MBI named the Caring Mindfulness-based Approach for Seniors (CMBAS) and an HSMP. The study was conducted across four sites (London, Cologne, Lyon, and Barcelona). The trial was registered on ClinicalTrials.gov (NCT03005652). SCD-Well was sponsored by the French National Institute of Health and Medical Research (INSERM), and ethical approval and regulatory authorizations were obtained at each site. Written informed consent was obtained from all participants (please see the “Declarations” section for further details). Further details pertaining to the study’s eligibility criteria, interventions, and assessments are available in the trial protocol [21], as well as the primary outcome report, which focuses on trait anxiety [22].

### Procedure

Due to the group-based nature of the interventions, participants were recruited in two waves at each site. Briefly, participants fulfilling the eligibility criteria were invited to the baseline visit (week 0) for cognitive and behavioral assessments. They were then randomized with a 1:1 allocation, using permuted block sizes of 4 and 6, stratified by site and centralized via a secure electronic case report form. Participants were invited to meet their intervention facilitator at a pre-class meeting, during which their trial allocation was revealed. The assessments were repeated at both post-intervention (week 8) and 6-month (week 24) follow-up visits. The size of each intervention group ranged from 7 to 13 participants.

### Participants

Recruitment took place from March 2017 through January 2018. For study inclusion, participants were required to fulfill the research criteria for SCD [1]. Briefly, these require an individual to self-report a decline in cognitive function but to score normally on standardized cognitive tests used to screen for MCI and/or dementia. The SCD criteria exclude neurodegenerative diseases (except Alzheimer’s disease), psychiatric disorders, and clinically significant affective symptoms. However, subclinical affective symptoms are not exclusionary. All participants were recruited from memory clinics, and the minimum age for study eligibility was 60 years; these characteristics are associated with an increased risk of incident dementia in SCD [4].

### Interventions

#### Caring Mindfulness-based Approach for Seniors (CMBAS)

The CMBAS followed the general format of a mindfulness-based stress reduction program, consisting of a pre-class interview, eight weekly group-based sessions of 2 h, and a half-day of meditation practice in the sixth week of the program to help consolidate learning. In addition to standard MBI practices [11], CMBAS participants were also taught compassion meditation practices focusing on cultivating wholesome attitudes toward oneself and others. Additional modifications included the provision of psychoeducation designed to help participants with SCD deal more adaptively with cognitive concerns and a tendency to worry, building on earlier work by Zellner Keller et al. [23]. Participants were asked to engage in home practice for approximately 1 h per day on 6 days per week and to record whether they engaged in these practices in a diary. Home practice consisted of formal practices (e.g., following guided meditation audio recordings), as well as informal practices designed to help participants apply mindfulness skills to their daily lives (e.g., mindful eating—bringing awareness to the taste, smell, and texture of a meal).

#### Health Self-Management Program (HSMP)

The HSMP followed the same format and structure as CMBAS and was matched in administration, dosage, and duration (including a half-day review with a healthy lunch and a discussion in the sixth week of the program). The intervention was based on a manual for living with chronic health conditions [24]; the manual was available in English, French, Spanish, and German. A previous RCT of an MBI which included older adults with neurocognitive difficulties adapted the manual to be delivered as a group psychoeducation intervention [16]; the adapted program was used to equalize treatment expectancy between arms and control for the “non-specific” components of the MBI (e.g., social interaction, input from a professional facilitator and light physical activity). In the current trial, the topics taught in the HSMP included self-management, problem-solving, sleep, stress, exercise, managing medicines, communicating with family and healthcare professionals, eating, weight management, and planning for the future. To promote engagement, participants were asked to plan, undertake, and report back on weekly “action plans.” Implementation of “action plans” was recorded by participants in a diary.

### Intervention facilitators and psychometrists

Each site had two clinically trained facilitators experienced in leading group-based programs, one for each intervention. Facilitators received their respective intervention manual, instructions, and intervention-specific training prior to the start of the study. After each class, facilitators completed a self-report checklist [25] to indicate the extent to which they adhered to the session as outlined in the manual. They also received ongoing supervision to promote standardization of delivery across sites. All psychometrists were blind to participants’ allocation and completed the study-specific training in order to standardize the administration and scoring of outcome measures.

### Composite cognitive outcomes

We calculated three composite measures of cognition from the broad battery of tests that were administered (see Additional file 1: Supplementary Methods for details). Schneider and Goldberg [26] summarized the potential advantages of composite over individual cognitive measures, including greater sensitivity to detect cognitive changes and avoidance of ceiling and floor effects, improved test-retest reliability, and reduced statistical multiplicity. Furthermore, the wider breadth of composite (versus individual) cognitive measures reduces the chance that any performance gains simply reflect similarities between the intervention activities and outcome measures (primarily a concern for cognitive training interventions). Schneider and Goldberg noted that scores across various cognitive domains are correlated, and this justifies the creation of “global” composites; nevertheless, they also emphasized that the measurement of individual cognitive domains remains crucial [26]. We thus specified both a global and two domain-specific composites. The same statistical approach was used to create each composite (described in detail below for the global composite). Composite scores were only calculable for time points where participants had data available for all of the necessary constituent tests (for details of how missingness was handled, see the “Statistical analyses” section). For each of the three composites, higher scores reflect better performance. Following the calculation of the composites (see below), each had a mean of 0 but a standard deviation (SD) less than 1; composites were thus “re-standardized” prior to analyses.

#### Abridged Preclinical Alzheimer Cognitive Composite 5

Donohue and colleagues [27] devised a global composite comprising four cognitive tests (two episodic memory, one attention, and one dementia screening measure); the authors demonstrated that this measure was sensitive to amyloid-beta (Aβ)-related cognitive decline in four cohorts over a 36-month period. The composite was named the Preclinical Alzheimer’s Cognitive Composite (PACC) [27]. Subsequently, Papp and colleagues [28] demonstrated that the sensitivity of the PACC could be increased through the addition of a category fluency score; the revised five-item measure was designated the PACC5. We produced an “abridged,” four-item version of the PACC5 (PACC5_Abridged_) in SCD-Well, as only one episodic memory measure was available. The tests constituting the PACC5_Abridged_ were the Rey Auditory Verbal Learning Test (delayed recall), the WAIS-IV Coding subtest (raw score), category fluency for animals (total correct), and the Mattis Dementia Rating Scale-2 (total score). The primary cognitive functions assessed by these measures are episodic memory, attention, semantic fluency, and global neuropsychological status, respectively. To create the global composite, each constituent score was first standardized, by subtracting the baseline pooled sample mean from each individual’s score at each available time point, and the result was divided by the baseline pooled standard deviation. We then took the average of these four scores, yielding the PACC5_Abridged_.

#### Attention cognitive composite

We also calculated an attention cognitive composite (“attention composite”). To calculate this measure, we first standardized scores from the Trail-Making Test Part A (TMT-A; completion time in seconds), a “naming” condition from the Stroop requiring participants to name the color of rectangular stimuli arranged in a grid (completion time in seconds), and WAIS-IV Coding (raw score). TMT-A and Stroop scores were multiplied by minus one, so that higher scores reflected better performance. We took the average of these three standardized scores, yielding the attention composite.

#### Executive function cognitive composite

Lastly, we calculated an executive function cognitive composite (“executive composite”). To calculate this measure, we first standardized scores from the TMT-B (completion time in seconds), letter fluency for “P” (total correct), and a Stroop “interference” score (time in seconds). The Stroop interference score was calculated by subtracting the completion time of the Stroop naming condition (see the previous paragraph) from the completion time of a Stroop “incongruent” condition requiring participants to name the ink color of color words, where the ink color was incongruent with the word itself. TMT-B and Stroop interference scores were multiplied by minus one, so that higher scores reflected better performance. We took the average of these three standardized scores, yielding the executive composite.

### Additional measures

Depressive symptoms were assessed using the 15-item Geriatric Depression Scale (GDS-15; range 0–15); higher scores reflect greater depressive symptoms [29]. Anxiety was measured using the State-Trait Anxiety Inventory-State subscale (STAI-A; range 20–80); higher scores reflect greater anxiety [30]. After the first intervention session, each participant also completed the Credibility/Expectancy Questionnaire (CEQ), which measures participants’ perception of their assigned intervention’s credibility, as well as their associated degree of expectancy [31]. Responses were used to compare participants’ expectations and perceptions of interventional credibility between arms. At the final visit (V3) participants were asked whether they had continued practice during the preceding four weeks of the follow-up period.

### Statistical analyses

Sample size calculations were based on the expected effect size with 80% power and a two-sided type 1 error of 5% for the mean change in the SCD-Well primary outcome (i.e., STAI-Trait subscale) from pre- to post-intervention between intervention arms. This resulted in a minimum total number of 128 (64 per group) [21], which the trial exceeded (*n* = 147). For the present analyses, we calculated the statistical power for the PACC5_Abridged_ only, as this was considered the main outcome. For an effect size of 0.25 on the PACC5_Abridged_, the power achieved by the study was 33%; for an effect size of 0.50, the power was 87% (for the rationale for selecting these effect sizes and further details of the approach used to calculate power, please see Additional file 1: Supplementary Methods). Descriptive statistics were calculated for the sample’s demographics and baseline measures. Given participants were randomized to interventions, we did not test for demographic or baseline differences between arms [32]. Here, we focus on the results for the three cognitive composites; data and models for individual cognitive tests are provided in Additional file 1: Tables S2-S3 and Figs. S2-S3. Linear mixed models (LMMs) were used to assess the effect of intervention assignment on outcomes over time. For each LMM, all participants who had at least one score for the respective outcome were included. All models included fixed effects for age at baseline (years), education level (years), baseline STAI-A score, baseline GDS-15 score, sex, and study site, as well as random participant intercepts. The parameters time (in weeks), trial arm, and the interaction between time and trial arm were also included to ascertain whether (a) outcome scores changed during the trial and (b) any observed change differed by arm. The use of a continuous-time metric (i.e., weeks) linearly constrained the modeled cognitive trajectories. Given other trajectories were plausible (e.g., improvement during the intervention period, but not during follow-up), we also analyzed the outcomes using LMMs with a factorial time metric (i.e., using the visit structure: baseline, post-intervention, and follow-up).

**Fig. 1:**
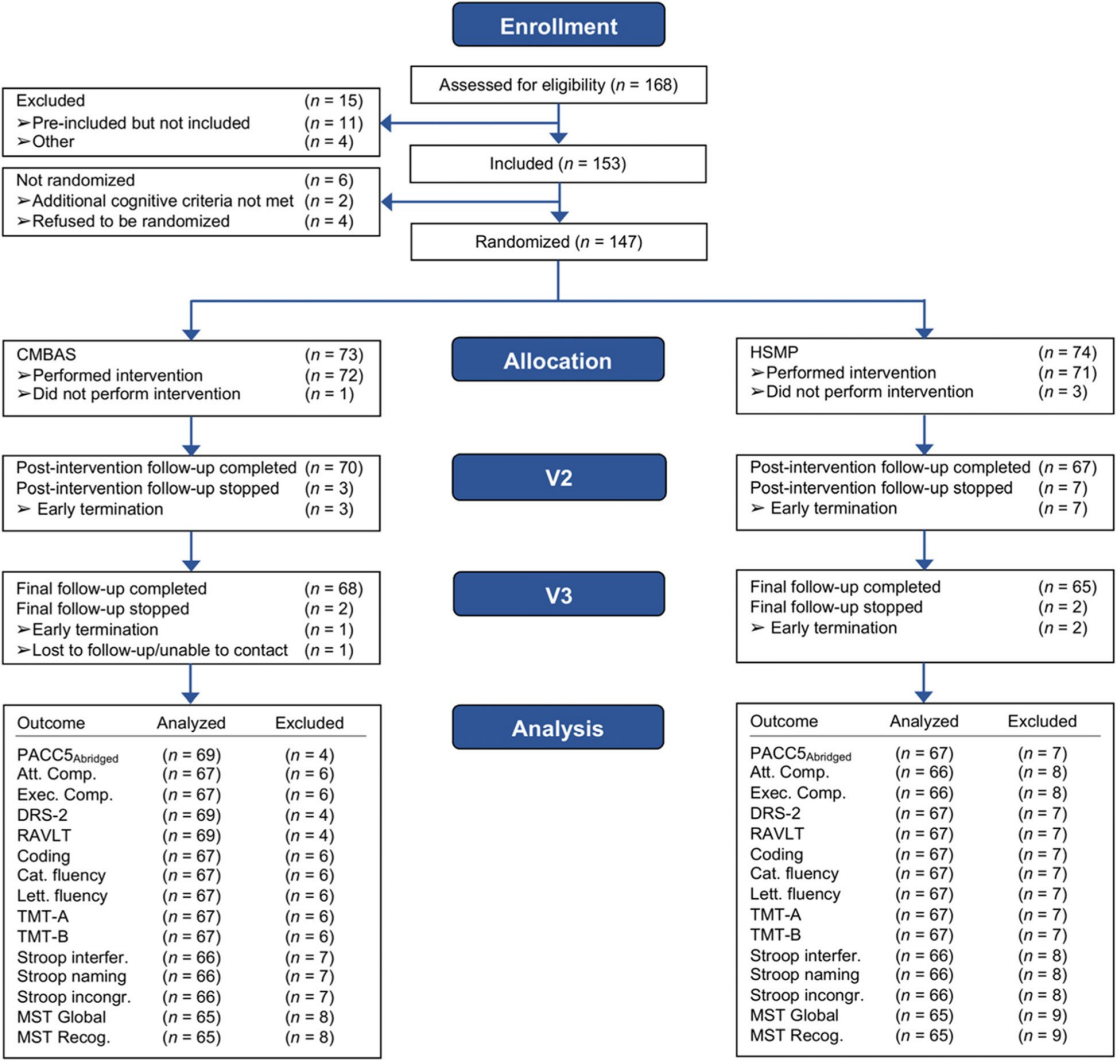
Consort flow diagram of enrollment and randomization to CMBAS and HSMP. The *n*s analyzed and excluded reflect the PP analyses. “Analyzed” participants were those with ≥ 2 observations for the respective measure (i.e., used to estimate the change in the outcome). While the LMMs also included participants who had baseline data only, these data were used solely for the estimation of intercepts (see Additional file 1: Table S2 for *n*s with non-missing baseline observations). CMBAS, Caring Mindfulness-Based Approach for Seniors; HSMP, Health Self-Management Program; V2, post-intervention; V3, follow-up; PP, per-protocol; PACC5_Abridged_, Abridged Preclinical Alzheimer Cognitive Composite 5; Att. Comp., attention composite; Exec. Comp, executive composite; DRS-2, Mattis Dementia Rating Scale-2; RAVLT, Rey Auditory Verbal Learning Test; Coding, Wechsler Adult Intelligence Scale-IV Coding; Cat. fluency, category fluency; Lett. fluency, letter fluency; TMT, Trail-Making Test; Stroop interfer., Stroop interference; Stroop incongr., Stroop incongruent; MST Recog., Mnemonic Similarities Task Recognition

Analyses were conducted according to both per-protocol (PP) and intention-to-treat (ITT). In the “Results” section we report PP analyses and note where these differ from ITT. The PP analyses included all available (i.e., non-missing) cognitive test data; the main reasons for missingness were participants not attending the post-intervention and/or follow-up visits due to dropping out or being lost to follow-up (see Fig. 1 for the CONSORT flow diagram). In addition, a sensitivity analysis re-estimated all PP LMMs using only the subset of participants who attended ≥ 4 intervention sessions; these analyses were motivated by previous research adjudging four MBI sessions to be an adequate minimal dose [33]. A series of PP linear regression analyses were conducted to determine the strength of association between participant baseline characteristics (i.e., predictors) and change on each composite in each trial arm separately. The outcome (i.e., dependent variable) for analyses was the follow-up (week 24) minus the baseline (week 0) score. The candidate predictors included in separate regression models were age, sex, education, site, GDS-15, STAI-A, CEQ-credibility, CEQ-expectancy, and the baseline score on the respective composite. All models controlled for age, sex, education, and site (either through the inclusion of these as the predictor of interest or as covariates).

For the ITT analyses, missing outcome data (for participants who dropped out or were lost to follow-up) were multiply-imputed using chained equations (the missing data pattern is presented in Additional file 1: Fig. S1). Given participants were randomized after their first cognitive assessment, virtually all baseline data were available for inclusion in the imputation models. Five datasets were “completed” using multiple imputation, and the LMM for each outcome was estimated using each of these five datasets. Finally, the five iterations of each LMM were pooled to yield a single ITT model for each outcome (for full details, see Additional file 1: Supplementary Methods).

Analyses were conducted in *R* v.4.0.2 under *RStudio* v.1.3.1073. LMMs were fit using the package *lme4* v.1.1-27.1; *p*-values for LMMs were obtained via *lmerTest* v.3.1-3. LMM-adjusted means and 95% confidence intervals (CIs) for each arm/outcome/time point, as well as change (Δ) in composite scores within and across groups, were produced using *emmeans* v.1.7.0. Multivariate imputation by chained equations was performed using *mice* v.3.14.0. For all analyses, uncorrected *p*-values are reported and were deemed statistically significant at < 0.05.

### Cognitive retest effects

Individuals undergoing repeated cognitive testing on the same measures are likely to learn task characteristics, which may result in improved performance over time. This study did not include an inactive comparator condition, and thus, cognitive retest effects could not be quantified empirically; we thus adjusted for these in statistical analyses. Cognitive retest effects were modeled based on recommendations [34]. Among the three strategies available, we utilized the first approach (referred to by the authors as “jump”); this specification was selected as the two alternatives were highly collinear with time (see Additional file 1: Supplementary Methods and Table S1 for details). This approach engenders the inclusion of a time-varying LMM covariate taking the value of “0” at baseline and “1” at the two subsequent visits. This coding represents participants’ lack of prior experience with the cognitive tests at baseline and their increased familiarity with these at weeks 8 and 24. The process of deciding which of the three cognitive retest effect specifications to use is described in Additional file 1: Supplementary Methods. The chosen cognitive retest effect covariate (coded as “0,” “1,” “1”) was only included in LMMs using linear time (i.e., weeks 0, 8, 24); both the effects of time and cognitive retesting could be estimated in these models. However, the cognitive retest effect parameter was not estimable (and thus not included) in LMMs using factorial time (i.e., according to visit).

## Results

Data collection was completed on September 18, 2018. A total of 147 participants with SCD (mean age 72.7 ± 6.9 years; 64% female) were randomized. See Table 1 for the sample baseline characteristics and Fig. 1 for the CONSORT flow diagram. The number of participants in each arm with data available for each outcome/time point is displayed in Additional file 1: Table S2.

**Table 1:** Sample baseline characteristics

	CMBAS (***n*** = 73)	HSMP (***n*** = 74)
Recruitment site (*n*, %)		
London, UK	14 (19)	14 (19)
Lyon, France	20 (27)	20 (27)
Cologne, Germany	19 (27)	20 (27)
Barcelona, Spain	20 (27)	20 (27)
Sex (female; *n* (%))	47 (64)	48 (65)
Ethnicity (white; *n* (%))	69 (95)	72 (99)
Age (years; *x̅* ± SD)	72.1 ± 7.5	73.2 ± 6.2
Education (years; *x̅* ± SD)	13.9 ± 3.8	13.4 ± 3.4
MMSE (*x̅* ± SD)	28.7 ± 1.2	28.9 ± 1.0
PACC5_Abridged_ (*x̅* ± SD)^a^	0.05 ± 1.05	− 0.05 ± 0.96
Attention composite (*x̅* ± SD)^b^	0.04 ± 1.10	− 0.01 ± 1.03
Executive composite (*x̅* ± SD)^c^	− 0.01 ± 1.01	0.01 ± 1.00
STAI-A (*x̅* ± SD)^d^	33.6 ± 9.8	31.6 ± 8.4
GDS-15 (*x̅* ± SD)^d^	3.1 ± 2.5	2.0 ± 2.0

*Abbreviations*: *x̅* mean, *SD* standard deviation, *CMBAS* Caring Mindfulness-Based Approach for Seniors; *HSMP* Health Self-Management Program, *PACC5*_*Abridged*_ Abridged Preclinical Alzheimer Cognitive Composite 5, *MMSE* Mini-Mental State Examination, *STAI-A* State-Trait Anxiety Inventory-State Subscale, *GDS-15* Geriatric Depression Scale

^a^*n* = 145

^b^*n* = 144

^c^*n* = 142

^d^*n* = 146

### Intervention fidelity

In the CMBAS condition, checklists indicated that 87.5% of sessions included all planned elements, with facilitators missing no more than one element in a session. All missed elements were minor in nature (e.g., shortening of movement practices due to time constraints). In the HSMP condition, checklists indicated that facilitators covered all planned elements without exception.

### Interventional credibility, expectancy, and engagement

No significant differences were observed between the trial arms for mean (SD) CEQ-credibility (CMBAS = 5.9 ± 2.2; HSMP = 5.3 ± 1.9) or CEQ-expectancy (CMBAS = 4.5 ± 1.9; HSMP = 4.1 ± 1.8). Similarly, there were no significant between-arm differences for the mean number of intervention sessions attended (CMBAS = 6.7 ± 2.8; HSMP = 6.8 ± 2.7), the proportion of participants who attended ≥ 4 intervention sessions (CMBAS = 81%; HSMP = 85%), or the proportion of participants who reported continued engagement with CMBAS/HSMP activities between the post-intervention (week 8) and follow-up (week 24) visits (CMBAS = 59%; HSMP = 54%). Furthermore, one hundred six (72%) participants completed home practice on at least four occasions (CMBAS = 55 [75%]; HSMP = 51 [69%]; these proportions did not significantly differ).

### Composite cognitive outcomes

#### PACC5_Abridged_

**Fig. 2:**
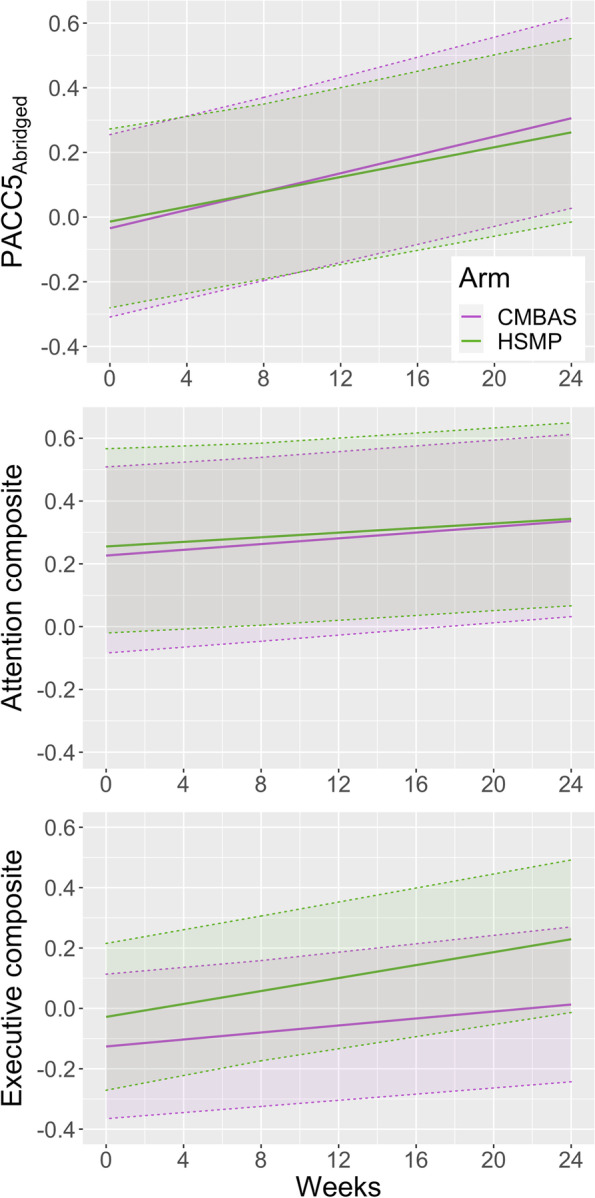
Estimated change in cognitive composite scores for each trial arm. The graphs visualize the trajectories modeled using the PP linear time LMMs. The cognitive retest effect parameters were omitted from the graphed models, as these resulted in discontinuous trajectories. The time-by-arm interaction was not significant for any composite (*p*s > 0.29), although PACC5_Abridged_ scores increased in both arms during the trial (*p* < 0.001). In order to aid interpretability, the graphed data are for a “prototypical” female participant with sample grand mean values for age, education, state anxiety, and depressive symptoms, at the Barcelona site. Shaded areas are 95% confidence intervals for the fixed effects. *Abbreviations*: PACC5_Abridged_, Abridged Preclinical Alzheimer Cognitive Composite 5; CMBAS, Caring Mindfulness-Based Approach for Seniors; HSMP, Health Self-Management Program; LMM, linear mixed model; PP, per-protocol

Findings from the PP and ITT models for the PACC5_Abridged_ were equivalent; the following results describe the PP analyses (for ITT models, see Additional file 1: Table S5). The LMM using a linear time metric (i.e., weeks) showed a statistically significant increase in PACC5_Abridged_ scores overall during the study (Δ [95% CI] = 0.31 [0.21, 0.41]). The interaction between time and trial arm was non-significant, indicating that trajectories did not differ between arms (CMBAS = 0.34 [0.17, 0.51]; HSMP = 0.28 [0.10, 0.45]). The LMM using a factorial time metric (i.e., visits) revealed that, while PACC5_Abridged_ performance did not significantly change between baseline and post-intervention (week 8), scores significantly increased from baseline to follow-up (0.32 [0.22, 0.42]). The visit-by-arm interaction was not significant at post-intervention nor follow-up. The improvement in PACC5_Abridged_ at follow-up was thus comparable in both arms (CMBAS = 0.34 [0.19, 0.48]; HSMP = 0.30 [0.15, 0.44]). These findings were substantively unchanged in sensitivity analyses. Table 2 shows the PP LMM coefficients of interest for the PACC5_Abridged_ and other composites; these data are presented visually in Fig. 2.

**Table 2:** Change in cognitive composite scores during the study

Composite	LMM coefficients (linear time specification)		LMM coefficients (factorial time specification)	
	Parameter	Estimate [95% CI]	Parameter	Estimate [95% CI]
PACC5_Abridged_			Post-intervention visit	0.04 [− 0.01, 0.10]
	Time (weeks)	**0.12 [0.05, 0.18]**	Follow-up visit	**0.16 [0.10, 0.22]**
	Time × arm	− 0.02 [− 0.09, 0.04]	Post-intervention × arm	0.04 [− 0.02, 0.11]
	Practice	0.04 [− 0.02, 0.09]	Follow-up × arm	− 0.01 [− 0.08, 0.05]
Attention composite			Post-intervention visit	0.05 [− 0.01, 0.10]
	Time (weeks)	0.02 [− 0.04, 0.08]	Follow-up visit	**0.06 [0.01, 0.11]**
	Time × arm	− 0.01 [− 0.07, 0.05]	Post-intervention × arm	− 0.01 [− 0.07, 0.05]
	Practice	0.04 [− 0.01, 0.09]	Follow-up × arm	− 0.01 [− 0.07, 0.05]
Executive composite			Post-intervention visit	0.05 [− 0.03, 0.12]
	Time (weeks)	0.03 [− 0.06, 0.11]	Follow-up visit	0.07 [− 0.00, 0.15]
	Time × arm	0.04 [− 0.04, 0.13]	Post-intervention × arm	0.02 [− 0.06, 0.11]
	Practice	0.04 [− 0.03, 0.11]	Follow-up × arm	0.05 [− 0.04, 0.13]

The model fits presented in the table are PP analyses. Regression coefficients are standardized. The time metric for linear time models was weeks (continuous) and for factorial time models, visits (factor). For factorial time models, the reference visit is the baseline. The post-intervention visit was at week 8, and the follow-up visit was at week 24. For both types of model, the reference trial arm is HSMP; positive coefficients for the interaction terms thus represent a relatively greater improvement in the HSMP (vs. CMBAS) arm; negative coefficients indicate the converse. Coefficient estimates in bold had *p*-values < 0.05 in the initial models. All models were adjusted for sex, age, years of education, state anxiety, depressive symptoms, and trial site; models using the linear time specification were also adjusted for cognitive retest effects. None of the models was substantively altered in sensitivity analyses which only included participants who attended ≥ 4 intervention sessions

*Abbreviations*: *PACC5*_*Abridged*_ Abridged Preclinical Alzheimer Cognitive Composite 5, *CMBAS* Caring Mindfulness-Based Approach for Seniors, *HSMP* Health Self-Management Program, *CI* confidence interval, *LMM* linear mixed model, *PP* per-protocol

#### Attention composite

The linear time LMM did not show an effect of time on attention composite scores in either the PP or ITT analyses, neither was there a significant interaction between time and trial arm. The factorial time LMM did not show a significant change for this outcome between baseline and post-intervention (week 8) in PP analyses, but the ITT model showed a significant improvement over this interval. Moreover, both PP and ITT analyses showed that attention scores increased overall from baseline to follow-up visit (0.11 [0.02, 0.20]). The visit-by-arm interaction was not significant at post-intervention nor follow-up in either analysis. Considered separately, the within-group change in attention composite scores from baseline to follow-up was not significant for either arm (CMBAS = 0.12 [− 0.01, 0.25]; HSMP = 0.10 [− 0.04, 0.23]). These findings were substantively unchanged in sensitivity analyses.

#### Executive composite

The PP and ITT analyses yielded equivalent findings for the executive composite. The linear time LMM did not show an effect of time on executive composite scores, neither was there a significant interaction between time and trial arm. The results from the factorial time LMM supported these findings; scores on the executive composite did not increase from baseline to post-intervention (week 8), nor from baseline to follow-up (week 24). There were no significant interactions with the trial arm. These findings were substantively unchanged in sensitivity analyses.

### Predicting response to interventions

Analyses (according to PP) were conducted using linear regression to determine the strength of association between participant baseline characteristics and change on each composite during the study (for each arm separately). The candidate predictors were age, sex, education, site, GDS-15, STAI-A, CEQ-credibility, CEQ-expectancy, and the baseline composite score. Considering the PACC5_Abridged_, in the CMBAS arm only, female (versus male) sex predicted significantly greater PACC5_Abridged_ gains; higher CEQ-credibility ratings were also associated with greater increases in global cognition in CMBAS participants. For the attention composite, lower baseline scores in the CMBAS arm were associated with greater gains on this measure. HSMP participants at the Lyon (versus Barcelona) site also showed greater attentional improvement. For the executive composite, lower baseline scores in both arms were associated with greater gains. Lower GDS-15 scores in the CMBAS arm were also associated with greater executive composite gains. See Additional file 1: Table S4 for further details.

## Discussion

SCD-Well was a large, multicenter RCT that randomized individuals with SCD to one of two 8-week non-pharmacological interventions. Here, we report outcome data for three composites, measuring global cognition (i.e., PACC5_Abridged_), attention, and executive functioning. Scores on the PACC5_Abridged_, a measure previously shown to be sensitive to early Aβ-related cognitive decline [27, 28], improved in both arms from baseline to follow-up (week 24), but improvements did not differ between arms. The magnitude of the increase in PACC5_Abridged_ scores corresponded to a small effect size (CMBAS, 0.34; HSMP, 0.30). These results were unchanged for the subset of participants who attended four or more intervention sessions. Therefore CMBAS, like other MBIs [14], improved global cognition, but not more than a health self-management comparator.

Scores on the attention composite did not improve in the statistical model using linear time, but scores improved at post-intervention (ITT only) and follow-up (both PP and ITT) in the factorial time models. A possible explanation for this discrepancy is that the adjustment for cognitive retest effects (not possible in the factorial time model due to statistical constraints) attenuated effects in the linear time model. While some of the analyses using factorial time showed an increase in attention scores overall, none indicated improvement for either arm individually (i.e., within groups). For example, the baseline to follow-up analyses showed significant attentional improvement overall, but not for either arm separately. This suggests that the within-group analyses may have been underpowered. In summary, on the basis of the mixed findings reported above, we conclude that there was weak evidence of an effect of both interventions on attention. Neither linear nor factorial time models identified an effect of either intervention on the executive composite.

To support the interpretation of our findings, we considered the results from recent meta-analyses which pooled cognitive data from MBI RCTs. While a number of quantitative syntheses exist, some excluded older adults (e.g., [35]), did not report results for younger and older adults separately (e.g., [36]) and/or included non-randomized studies (e.g., [37]). In the following discussion, we thus focus on the two meta-analyses which reported data from older adult RCTs separately (or exclusively) [14, 20]. One of the reviews reported that MBIs outperformed comparators in an analysis combining outcomes across domains [14]. We thus hypothesized that the current MBI would outperform the HSMP for the PACC5_Abridged_, given the various cognitive functions assessed by its constituents. Contrary to our prediction, PACC5_Abridged_ scores improved to a similar degree in both trial arms. Returning to the prior meta-analysis, half of the comparators included in the quantitative synthesis were inactive, and subgroup analyses suggested that the overall effect was driven by results from inactively controlled trials [14]. Integrating our findings with those of the meta-analysis, CMBAS—in common with other MBIs—improved global cognition but not to a greater extent than an active comparator.

Theoretical frameworks (both general [38] and aging-specific [39, 40]) posit that engagement with regular mindfulness practice confers gains to attention and executive function. It is thus unsurprising that a growing number of older adult MBI studies include outcome measures that assess these cognitive domains. Beginning with attention, we observed weak evidence of a positive effect across both arms. A previous RCT with SCD participants reported that an MBI outperformed a health education program for a measure of attention regulation (intraindividual variation in reaction time on a go/no-go task), although improvements in task accuracy were observed in both arms [15]. Lastly, two quantitative syntheses both concluded that MBIs did not outperform comparators for improving attention outcomes in older individuals [14, 20]. The present findings are thus broadly in line with earlier work.

Considering the executive function, the lack of an effect in the CMBAS arm runs contrary to our hypothesis, namely, a meta-analysis of MBI RCTs reported a significant effect in this domain in older adults [14]. The meta-analysis also examined the effects of MBIs on subdomains of executive function (inhibition, task switching, and working memory); the only subdomain to improve (across all age groups, as there were insufficient data to analyze older adults separately) was working memory [14]. The executive composite used in our trial included measures of inhibition and task switching, but none gauging working memory. If MBIs improve working memory specifically, rather than executive function generally, the lack of measures of the former in this trial may account for the discrepancy. A different meta-analysis—predominantly comprising actively controlled RCTs—found that, relative to comparators, MBIs did not improve executive function in older persons [20]. The disconfirmation of our executive function hypothesis may thus be explicable in terms of the specific outcomes and/or comparator types used in this versus earlier research.

It is important to consider the potential contribution of cognitive retest effects to the current results. Because this trial did not include an inactive comparator (e.g., a waitlist), we were unable to quantify cognitive retest effects empirically. When we controlled for these statistically we continued to observe increases in PACC5_Abridged_ scores, suggesting that the interventions were, indeed, conferring benefits to global cognition. Moreover, a recent review concluded that worse baseline cognition was associated with smaller cognitive retest effects [41], whereas the present study observed that worse baseline cognitive performance was associated with greater improvement during the study. Considering the above evidence, it seems unlikely that the present increase in PACC5_Abridged_ in both trial arms can be satisfactorily accounted for by cognitive retest effects alone.

Two types of mechanism, shared and specific, may account for the intervention-related improvements in PACC5_Abridged_. The first relates to the interventional elements common to both the CMBAS and HSMP; these include increased social contact, gentle exercise, behavioral activation, and input from caring professionals [42, 43]. Participants’ anticipation of benefit is another factor which can contribute to experimental effects [44]. The second type of mechanism relates to the elements unique to each intervention. For the CMBAS, the core element is the teaching of mindfulness and compassion-focused meditation practices; these are hypothesized to strengthen attention control, metacognitive monitoring, and prosocial capacities [39, 40]. The HSMP “curriculum” was considerably more varied, featuring a diverse array of topics, and participants implemented personalized action plans based on their unique goals. These characteristics make it more difficult to directly attribute PACC5_Abridged_ gains to specific interventional elements. Nevertheless, considering the topics taught in the HSMP [24], possible mechanisms driving PACC5_Abridged_ gains include improved sleep [45], increased physical activity [46], and/or a healthier diet [47]. In the context of RCTs, an intervention must outperform an active comparator for its effects to be unambiguously attributed to intervention-specific mechanisms [43]. While intervention-specific mechanisms may have been one factor which contributed to the observed cognitive gains, the present results do not provide strong evidence for this.

The present research has a number of strengths. SCD-Well remains one of a limited number of RCTs which recruited individuals with SCD, achieved a larger sample size than comparable studies, used blinded outcome raters, and included an active comparator which matched the MBI on a number of key characteristics. Moreover, the study measured the outcomes at both post-intervention (week 8) and follow-up (week 24), administered a comprehensive battery of cognitive measures across a range of domains, and is one of the first reported RCTs to include a version of the PACC as an outcome. The study thus addressed a number of limitations noted in previous reviews of the MBI [48] and SCD non-pharmacological interventions literature [9, 10]. Moreover, all participants were identified via memory clinics and were aged 60 years and above. These factors are associated with greater dementia risk in SCD [4], and our findings thus speak directly to the contemporary imperative to prevent cognitive decline [49]. Lastly, we considered the role of cognitive retest effects and statistically adjusted for these in line with published guidelines [34]. While we could not rule out cognitive retest effects, the balance of evidence suggests that the currently observed improvements are, at least in part, attributable to the interventions.

The study also has limitations. Firstly, the data reported here were secondary outcomes of the SCD-Well RCT, and we did not correct statistical models for multiple comparisons. Given the increasing interest in MBIs as a novel strategy to reduce cognitive decline in older persons [39, 40], it will be important for future trials to specify cognitive measures as primary outcomes; this will avoid statistical multiplicity and ensure that sufficient power is available to detect cognitive changes. Furthermore, while evaluating cognitive trajectories is more practicable than measuring dementia incidence, trials demonstrating cognitive effects (such as this one) require a confirmation from studies using clinically meaningful endpoints [50]. Considering the interventions, the home practice assigned to participants differed between CMBAS and HSMP (reflecting the interventions’ distinct rationales and themes). For CMBAS, the home practices were relatively fixed and prescribed by the facilitator, whereas participants in HSMP devised their own action plans based on their own goals. This difference diminished the equivalence of the interventions and may have influenced the findings. Improving the similarity of home practice assignments across intervention arms will be an important consideration for future trials. Moreover, CMBAS and HSMP were relatively brief; longer interventions may be necessary to maintain salutary effects over an extended time period. However, over 50% of participants in both arms reported continued engagement with intervention activities between the post-intervention and follow-up visits, and cognition continued to improve during this period; there was thus some evidence that the interventions had enduring effects. The vast majority of our participants were white; this homogeneity may limit the generalizability of this research to other groups, as clinical presentation and therapeutic response may vary by ethnicity [51]. Lastly, the absence of a working memory measure in the present study prevented an evaluation of MBI effects on this domain in SCD; the inclusion of such a measure is recommended for future MBI studies targeting cognitive decline.

In conclusion, we studied the effects of two non-pharmacological interventions, based on mindfulness and health self-management respectively, on a range of cognitive outcomes in older adults with SCD. Both interventions conferred small, non-differing, and significant improvements to the PACC5_Abridged_, a composite sensitive to Aβ-related decline; gains were maintained for at least 4 months post-intervention. In contrast, there was weaker evidence for salutary effects across both arms on an attention composite, and no effect on executive function. Integrating both the current and previous research findings, cognitive retest effects may have contributed to the observed gains, but could not account for these entirely. These results are encouraging and add to the recognized benefits of MBIs on psycho-affective outcomes in older adults [52]. Future investigators are encouraged to evaluate MBIs of longer durations, implement rigorous control for cognitive retest effects [53], seek to identify which interventional components may be driving results, and evaluate if improved cognitive function translates to a subsequent reduction in dementia incidence.

## Supplementary Information

**Additional file 1. **Supplementary Methods. Supplementary Results. **Table S1**. Collinearity diagnostics for alternate specifications of the cognitive retest effect variable. **Table S2**. Unadjusted (observed) and model-adjusted means for each trial arm at each timepoint (composite and individual cognitive outcomes). **Table S3**. Linear mixed models fitted using a linear and factorial time specification, respectively (individual outcomes only – see main paper Table 2 for composite outcomes). **Table S4**. Association between candidate predictors and baseline (week 0) to follow-up (week 24) change scores on composite outcomes, broken down by trial arm. **Table S5**. Pooled linear mixed models derived from analyses of multiply-imputed SCD-Well trial data (m = 5). **Figure S1**. Missing data pattern for the ‘wide’ format dataset. **Figure S2**. Estimated change in individual cognitive tests for each trial arm (linear-time specification). **Figure S3**. Estimated change in individual cognitive tests for each trial arm (linear-time specification).

## Data Availability

The datasets used and/or analyzed during the current study are available from the corresponding author on reasonable request, subject to approval by the project executive committee and study sponsor. To gain access, researchers will need to submit a data request form.
