# Supplementary material for: Effects of a mindfulness-based versus a health self-management intervention on objective cognitive performance in older adults with subjective cognitive decline (SCD): a secondary analysis of the SCD-Well randomized controlled trial
Source: Alzheimers Res Ther. 2022 Sep 6;14:125. doi: 10.1186/s13195-022-01057-w (PMC9446839; doi:10.1186/s13195-022-01057-w)
Supplement: Supplementary file 1 — Additional file 1. Supplementary Methods. Supplementary Results. Table S1. Collinearity diagnostics for alternate specifications of the cognitive retest effect variable. Table S2. Unadjusted (observed) and model-adjusted means for each trial arm at each timepoint (composite and individual cognitive outcomes). Table S3. Linear mixed models fitted using a linear and factorial time specification, respectively (individual outcomes only – see main paper Table 2 for composite outcomes). Table S4. Association between candidate predictors and baseline (week 0) to follow-up (week 24) change scores on composite outcomes, broken down by trial arm. Table S5. Pooled linear mixed models derived from analyses of multiply-imputed SCD-Well trial data (m = 5). Figure S1. Missing data pattern for the ‘wide’ format dataset. Figure S2. Estimated change in individual cognitive tests for each trial arm (linear-time specification). Figure S3. Estimated change in individual cognitive tests for each trial arm (linear-time specification). [file 13195_2022_1057_MOESM1_ESM.docx]

**Supplementary materials for:** Effects of a mindfulness-based intervention versus health self-management on cognitive performance in older-adults with subjective cognitive decline (SCD): The SCD-Well randomized superiority trial

Table of Contents

[Supplementary Methods 2](#_Toc109304042)

[Outcome measures 2](#_Toc109304043)

[Individual cognitive outcome measures 2](#_Toc109304044)

[Statistical analyses 3](#_Toc109304045)

[Specification of the cognitive retest effect variable 3](#_Toc109304046)

[Evaluating collinearity between the cognitive retest effect and linear-time parameters 3](#_Toc109304047)

[Intention-to-treat analysis using multiple imputation 4](#_Toc109304048)

[Power analysis 6](#_Toc109304049)

[Supplementary Results 7](#_Toc109304050)

[Per-protocol analyses 7](#_Toc109304051)

[Cognitive test outcome data 7](#_Toc109304052)

[Fitted linear mixed models 9](#_Toc109304053)

[Predicting response to interventions 11](#_Toc109304054)

[Intention-to-treat analyses 14](#_Toc109304055)

[Fitted linear mixed models 14](#_Toc109304056)

[References 16](#_Toc109304058)

List of tables

[**Table S1** Collinearity diagnostics for alternate specifications of the cognitive retest effect variable 4](#_Toc109304100)

[**Table S2** Unadjusted (observed) and model-adjusted means for each trial arm at each timepoint (composite and individual cognitive outcomes) 7](#_Toc109304101)

[**Table S3** Linear mixed models fitted using a linear and factorial time specification, respectively (individual outcomes only – see main paper Table 2 for composite outcomes) 9](#_Toc109304102)

[**Table S4** Association between candidate predictors and baseline (week 0) to follow-up (week 24) change scores on composite outcomes, broken down by trial arm 11](#_Toc109304103)

[**Table S5** Pooled linear mixed models derived from analyses of multiply-imputed SCD-Well trial data (m = 5) 14](#_Toc109304104)

**List of figures**

**Figure S1** Missing data pattern for the ‘wide’ format dataset 6

**Figure S2** Estimated change in individual cognitive tests for each trial arm (linear-time specification) 13

**Figure S3** Estimated change in individual cognitive tests for each trial arm (linear-time specification) 14

# **Supplementary Methods**

## Outcome measures

### Individual cognitive outcome measures

*Mattis Dementia Rating Scale-2*

The Mattis Dementia Rating Scale-2 (DRS-2) is a standardized scale designed to quantify neuropsychological deficits in individuals presenting with cognitive difficulties [1]. The scale comprises five subscales (Initiation/Perseveration, Construction, Conceptualization, Memory and Attention) as well as a total score (range 0-144), which was the test metric analyzed in this study.

*Rey Auditory Verbal Learning Test*

The Rey Auditory Verbal Learning Test (RAVLT) evaluates verbal episodic memory in adults [2] via a list-learning paradigm. The participant is read 15 nouns, and is then asked to recall as many of these words as possible. This is repeated five times, after which a new list is read once and recall of items from that list is recorded. After this ‘interference’ trial, the participant is asked to recall the words from the first list. After a delay of approximately 20 minutes, the participant is again asked to freely recall the words from the first list (delayed recall; range 0-15); this was the RAVLT metric analyzed in this study.

*Wechsler Adult Intelligence Scale-IV Coding*

The Wechsler Adult Intelligence Scale-IV (WAIS-IV) Coding test evaluates attentional and associative learning capacities, as well as psychomotor speed [3, 4]. During WAIS-IV Coding the participant is first shown a key with the numbers 1 to 9. Each number is paired with a unique geometric symbol. The examinee is then shown ‘double’ rows of boxes containing numbers in the top row, and blank boxes below them. The task requires the participant to transcribe the appropriate geometric symbol under each number. The raw score is the number of correct items completed within 120 seconds (range 0-135).

*Verbal Fluency*

Two types of verbal fluency were measured (category and letter); both measures evaluate executive control and verbal ability [5]. For category fluency, individuals are required to generate as many animal words as possible within two minutes [6]. For letter fluency, individuals are required to generate as many words beginning with the letter ‘P’ as possible within two minutes [6]. For both tests, the total score (number of unique eligible responses) was analyzed.

*Trail-Making Test parts A and B*

Part ‘A’ of the Trail-Making Test (TMT-A) can be considered a test of simple visual attention and psychomotor speed, whereas part ‘B’ (TMT-B) assesses executive task switching. For TMT-A, participants are required to sequentially connect a series of encircled numbers scattered across the page [6]. For TMT-B, the encircled numbers are interspersed with encircled letters, and the participant must sequentially and alternately connect both the numbers and letters (e.g. *1* - *A* - *2* - *B*, etc.). For both parts, the time to completion (in seconds) was recorded. TMT scores were multiplied by minus one prior to analyses, so that higher scores reflected better performance.

*Stroop*

The Stroop test is a widely utilized test of executive inhibition. For each Stroop condition, stimuli were arranged in a 10 by 10 grid (i.e. 100 items in total) [6]. Across a series of three conditions, participants are required to name color swatches (printed in red, green, or blue; ‘Stroop naming’), read color names (printed in black; data not presently analyzed), or to name the color of the ink that color words are printed in, where this is incongruent with the word itself (‘Stroop incongruent’). ‘Stroop interference’ scores were also created for analyses, calculated by subtracting the completion time for Stroop naming from the equivalent score for Stroop incongruent [7]. Stroop scores were multiplied by minus one prior to analyses, so that higher scores reflected better performance.

*Mnemonic Similarities Task*

The Mnemonic Similarities Task (MST) is a computerized test of pattern separation – the process by which similar sensory inputs are orthogonalized into distinct, nonoverlapping representations, so that new memories can be stored without giving rise to excessive interference [8]. The MST consists of two phases: in the first phase, participants are shown pictures of everyday objects and must judge whether each image depicts an ‘indoor’ or an ‘outdoor’ object. Subsequently, participants perform an unexpected recognition memory test, whereby they must identify pictures as ‘old’, ‘similar’, or ‘new’. The stimuli for this phase comprise repeated, new, and similar images (i.e. targets, foils and lures) to those seen during the first phase. Discriminating a lure from the related, original image requires distinct representations – a hallmark of pattern separation. The MST comprises two scores – a global score calculated as the rate of ‘similar’ responses for lures minus ‘similar’ responses for foils (to account for any bias the participant has in using the ‘similar’ response), as well as a simple recognition score (target hits minus foil false alarms).

## Statistical analyses

### Specification of the cognitive retest effect variable

Cognitive retest effects (CREs) were modelled based on recommendations. CREs were only included in analyses using linear time. Vivot et al. [9] proposed a statistical adjustment to model CREs in studies of determinants of cognitive change in older adults. The authors proposed four different approaches to modelling CREs:

1. No CREs
2. *‘Jump’* - an indicator variable for the first cognitive visit, coded so that coefficients were positive for a boost in performance after initial testing (e.g., 0, 1, 1, . . ., 1)
3. *‘Hop’* - number of prior visits (e.g., 0, 1, 2, 3, . . .)
4. *‘Skip’* - square root of the number of prior visits (e.g., 0, 1, 1.4, 1.7, . . .)

We selected the optimal CRE specification for the PACC5_Abridged_ linear mixed model and then included this in the models for all the other cognitive outcomes, in order to maximize comparisons between these. We selected the optimal CRE specification according to the four step approach described by Vivot et al. [9](findings from our study colored blue):

1. First, choose an overall CRE structure by comparing the four models without including any exposure coefficient (i.e. including only time and the alternative CRE specifications). The presence of CREs can be tested by inspecting the *p*-value for the CRE variable. In the case of the presence of CREs, the best specification can be selected through goodness-of-fit criteria such as the AIC.

Across the three models including a CRE term (i.e. Jump, Hop, Skip), none had a statistically significant estimate for CREs (all *p*s ~ 0.2). Whilst evidence for the presence of CREs was lacking, we resolved to retain a CRE term in the model to control for this. Given that the AICs did not differ across the three models, we based our decision of which CRE term to include on the basis of the model with the least collinearity. Collinearity was assessed following guidelines available online [10] (see Table S1 for relevant collinearity diagnostics). The only CRE specification exhibiting acceptable collinearity was Jump, and we thus selected this CRE.

1. Second, after choosing the preferred CRE specification, model a 2-way interaction to assess whether the exposure of interest (in this case, trial arm) modifies the CREs. This model should include both the arm × CREs interaction and the arm × time interaction.

The arm × CREs interaction for the Jump model was not statistically significant (*p* = .08).

1. Third, if there is no evidence for an arm × CREs interaction, drop this term.

Based on our findings, we dropped the interaction.

1. Fourth, if there is an arm × CREs interaction, further investigation is required, as there are multiple potential reasons for this finding.

Not applicable.

### Evaluating collinearity between the cognitive retest effect and linear-time parameters

Each specification of time was assessed for collinearity with the time variable using Kappa (condition number) and Variance Inflation Factor (VIF) statistics (see Table S1). For the two multicollinearity diagnostics, the following recommended cut-offs were used for interpretation:

**Kappa:**

- < 10 is reasonable collinearity
- < 30 is moderate collinearity
- ≥ 30 is troubling collinearity

**VIF:**

- < 2.5 is acceptable
- It is recommended to investigate values ≥ 2.5
- Values over 5 are troubling

**Table S1** Collinearity diagnostics for alternate specifications of the cognitive retest effect variable

| **Practice specification (see [9])** | **Kappa** | **VIF** |
| --- | --- | --- |
| None (reference) | 2.88 | 1.00 |
| Jump* | 4.51 | 2.38 |
| Hop | 20.49 | 28.20 |
| Skip | 8.42 | 5.97 |

Abbreviation: *VIF* Variance Inflation Factor. *The only cognitive retest effect specification with acceptable Kappa and VIF statistics is ‘Jump’; this was thus selected for linear mixed model analyses using linear time.

### Intention-to-treat analysis using multiple imputation

An intention-to-treat (ITT) analysis was conducted on the full sample of 147 participants. Some outcome data were missing, and thus it was necessary to perform multiple imputation. Multivariate imputation by chained equations was performed using the *R* package *mice* v.3.14.0. The method used for the ITT analysis followed guidelines for multiply-imputing longitudinal data contained in a reference text [11]. Rather than imputing the composite variables, only the individual measures were initially imputed, and then the composites were created as per the method used for PP analyses. Identical to the PP analyses, all outcome measures were standardized prior to estimation of the linear mixed models. Specifically, the method comprised the following steps:

1. The study data were reshaped from ‘long’ to ‘wide’ format.
2. The missing data pattern was inspected and plotted (see Figure S1 below).
3. The potentially large number of predictors for multiple imputation was reduced via specifying a predictor matrix as per the reference text [11]. This involves limiting the variables used to impute missing data to those collected concurrently (i.e. at the same timepoint), except for the imputed variable collected at other timepoints. This avoids overdetermining – and reduces the time needed to complete – the imputations. In-line with recommendations, all variables used in the analysis model were included in the imputation model [12], except the ‘time’ and ‘cognitive retest effect’ variables; these variables were identical for all participants and the effect of time was already represented in the ‘wide’ format data via the inclusion of repeated measures.
4. On the basis of additional recommendations, missing data were imputed for each trial arm separately [12]. The rationale for this is that it preserves possible *time × arm* interaction effects on the outcome measures (i.e. the central quantity of interest).
5. Five datasets were imputed for each arm.
6. Each linear mixed model was estimated on these five datasets separately, and the results were then pooled (see Table S5).

Whilst the *mice* package for *R* is highly comprehensive, some advanced methods for linear mixed models are yet to be fully supported. There is a lack of support for making ‘predictions’ from pooled models; these were necessary to estimate bootstrapped confidence intervals for the figures showing the trajectories of each outcome during the trial. Moreover, neither the *emmeans* package (used to derive estimated marginal means from models), nor the function used to standardize linear mixed model coefficients were compatible with pooled models. Thus, the ITT results are only available in tabular format (i.e. no graphs were produced), no estimated marginal means were reported in the main Results, and (in contrast to the PP results) comprise unstandardized regression coefficients.

**Figure S1** Missing data pattern for the ‘wide’ format dataset

**
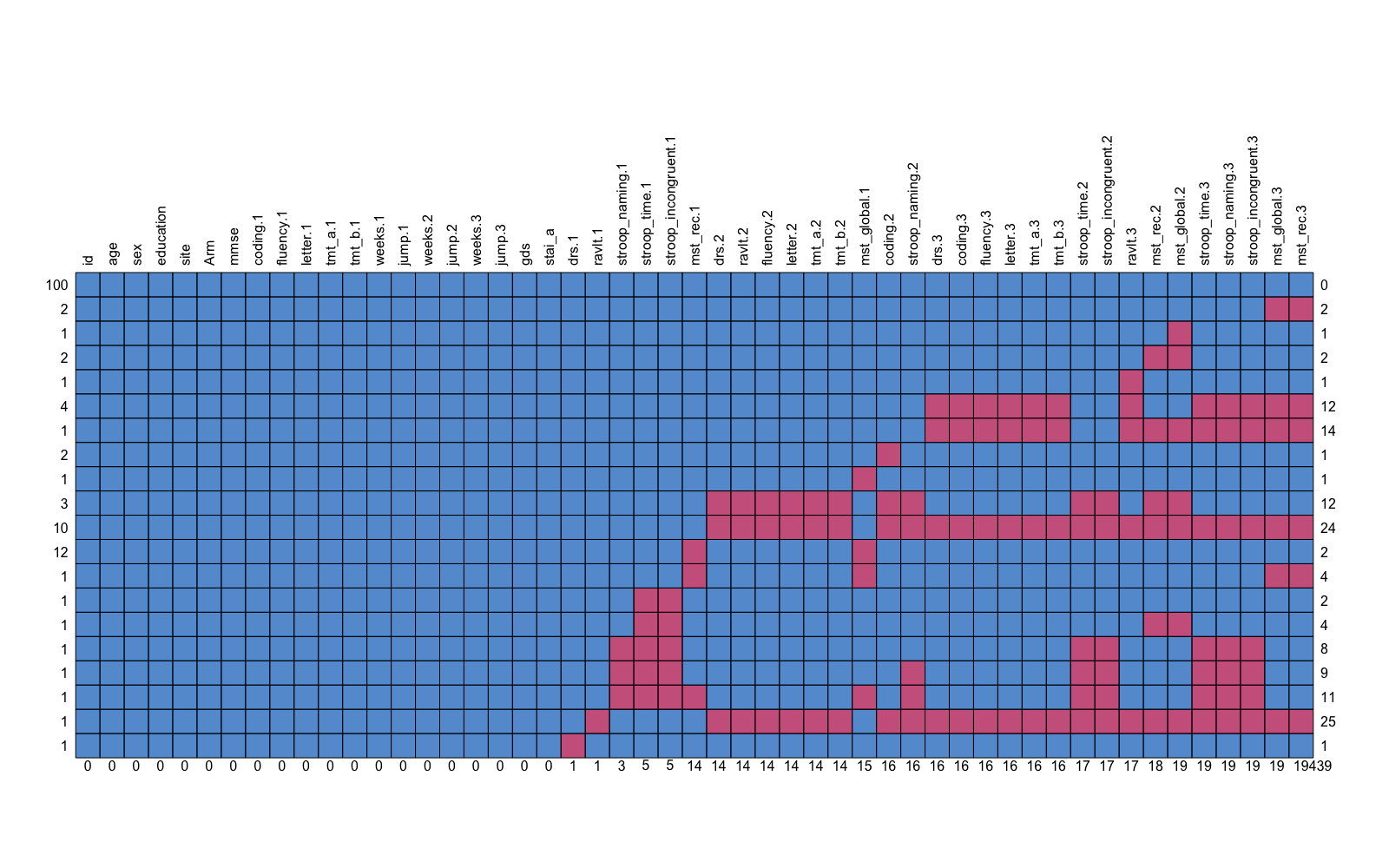
**

Missing data pattern for the ITT sample (*n* = 147), produced via the *md.pattern* function within the *mice* package for *R*. Each column represents a variable (note the ‘wide’ data format) with pink cells indicating missing data and blue cells observed data. The first column (left) provides the frequency (i.e. number of participants) of each pattern. The last column lists the number of missing entries per pattern. The bottom row provides the number of missing entries per variable, and the total number of missing cells. The ‘id’, ‘age’, ‘sex’, ‘education’, ‘site’, ‘Arm’, ‘mmse’, ‘gds’ and ‘stai_a’ variables were not time-varying. The remaining variables were time-varying outcome measures collected a maximum of three times; the number suffix indicates the respective visit. Abbreviations: *drs* Mattis Dementia Rating Scale-2; *ravlt* Rey Auditory Verbal Learning Test; *coding* Wechsler Adult Intelligence Scale-IV Coding; *tmt* Trail-Making Test; *mst* Mnemonic Similarities Task; *stroop_time* Stroop interference; *fluency* Category fluency; *letter* Letter fluency; *gds* 15-item Geriatric Depression Scale; *stai_a* State-Trait Anxiety Inventory-State subscale; *mmse* Mini-Mental State Examination; *ITT* Intention-to-treat.

### Power analysis

Given our use of linear mixed models (LMM) in the paper, it was necessary to conduct a simulation-based power analysis for the PACC5_Abridged_. The method we used followed published guidelines [13] (as well as the companion website [14]). Rather than performing a post-hoc power analysis using our dataset (for arguments against this, please see [15-17]), we opted to perform the power analyses using conventional/plausible effect size values. We chose two PACC5_Abridged_ between-arm effect sizes: 0.25 and 0.50. The first value corresponds to the cognitive deficit (on a global cognitive composite) reported in SCD versus healthy controls in a previous study [18]. The second effect size we used mirrored that harnessed for the primary outcome power analysis [19]. The power analyses thus addressed two conventional scenarios–the observation of ‘small’ and ‘medium’ effects, respectively.

We did not have access to a suitably-powered previous RCT dataset to base our estimation of the fixed effect coefficients and random effect variances for the simulation. We thus followed the guidelines [13] and artificially simulated a dataset for the power analyses. The artificial dataset had the same format and covariates as the ‘real’ dataset, but did not include any outcome data. Next, we specified the values for the fixed effect beta coefficients and random effect variances. In order to estimate these, we downloaded a freely-available dataset similar to that analyzed in the paper [20]. That study was also an RCT of a mindfulness-based program versus a health education program in older adults at risk of dementia, and measured interventional effects on cognitive outcomes at three timepoints. However, we opted to combine the external with an artificial dataset (rather than simply treating the present and online studies as equivalent) as the previous study randomized fewer participants than our study (and thus did not appear to fulfil the requirement of constituting ‘a preceding well-powered design’ [13]). For comparability with the current study, we created a global composite (comprising RAVLT delayed, color trails test interference, and semantic fluency) within the online dataset using the same methods as reported in the paper. At the final visit, 20% of participants in the external dataset had dropped out; we thus replaced their data using multiple imputation according to the methods outlined above. Following this, a linear mixed model (using factorial time, to minimize the number of parameters) was estimated on the external dataset using between-arm effect size values of 0.25 and 0.50. The coefficients and variances were then input into the *simr* v.1.0.6 package and combined with the artificial dataset to provide the PACC5_Abridged_ power estimates reported in the paper.

# **Supplementary Results**

## Per-protocol analyses

### Cognitive test outcome data

**Table S2** Unadjusted (observed) and model-adjusted means for each trial arm at each timepoint (composite and individual cognitive outcomes)

| **Measure/Visit** | **Sample size** | | **Unadjusted mean (SD)** | | **Adjusted mean [95% CI]** | |
| --- | --- | --- | --- | --- | --- | --- |
|  | **CMBAS** | **HSMP** | **CMBAS** | **HSMP** | **CMBAS** | **HSMP** |
| **PACC5_Abridged_** | | | | | | |
| Week 0 (baseline) | (*n* = 72) | (*n* = 73) | 0.05 (1.05) | -0.05 (0.96) | -0.07 [-0.35, 0.21] | -0.05 [-0.33, 0.23] |
| Week 8 (post-intervention) | (*n* = 65) | (*n* = 66) | 0.15 (1.05) | 0.15 (0.86) | 0.04 [-0.23, 0.31] | 0.04 [-0.23, 0.32] |
| Week 24 (follow-up) | (*n* = 66) | (*n* = 64) | 0.44 (1.06) | 0.26 (0.91) | 0.27 [-0.02, 0.56] | 0.23 [-0.07, 0.52] |
| **Attention composite** | | | | | | |
| Week 0 (baseline) | (*n* = 71) | (*n* = 73) | 0.04 (1.10) | -0.04 (0.90) | -0.03 [-0.33, 0.27] | 0.00 [-0.30, 0.29] |
| Week 8 (post-intervention) | (*n* = 63) | (*n* = 66) | 0.18 (0.96) | 0.06 (0.83) | 0.01 [-0.29, 0.30] | 0.03 [-0.26, 0.31] |
| Week 24 (follow-up) | (*n* = 65) | (*n* = 63) | 0.22 (1.05) | 0.09 (0.80) | 0.08 [-0.23, 0.39] | 0.09 [-0.22, 0.39] |
| **Executive composite** | | | | | | |
| Week 0 (baseline) | (*n* = 71) | (*n* = 71) | -0.01 (1.01) | 0.01 (1.00) | -0.10 [-0.37, 0.16] | -0.01 [-0.28, 0.26] |
| Week 8 (post-intervention) | (*n* = 65) | (*n* = 65) | 0.10 (0.90) | 0.13 (0.94) | -0.06 [-0.31, 0.19] | 0.08 [-0.17, 0.33] |
| Week 24 (follow-up) | (*n* = 65) | (*n* = 63) | 0.14 (0.99) | 0.27 (0.93) | 0.03 [-0.25, 0.32] | 0.25 [-0.04, 0.54] |
| **DRS-2 (Total)** | | | | | | |
| Week 0 (baseline) | (*n* = 72) | (*n* = 74) | 0.13 (1.03) | -0.12 (0.96) | -0.03 [-0.33, 0.27] | -0.10 [-0.40, 0.20] |
| Week 8 (post-intervention) | (*n* = 67) | (*n* = 66) | 0.02 (1.02) | 0.12 (1.02) | 0.05 [-0.22, 0.32] | -0.02 [-0.29, 0.25] |
| Week 24 (follow-up) | (*n* = 67) | (*n* = 64) | 0.33 (0.95) | 0.16 (0.99) | 0.20 [-0.13, 0.53] | 0.13 [-0.20, 0.46] |
| **RAVLT (Delayed recall)** | | | | | | |
| Week 0 (baseline) | (*n* = 73) | (*n* = 73) | -0.05 (1.06) | 0.05 (0.94) | -0.14 [-0.44, 0.15] | 0.04 [-0.25, 0.34] |
| Week 8 (post-intervention) | (*n* = 67) | (*n* = 66) | 0.23 (1.11) | 0.38 (0.92) | 0.00 [-0.28, 0.28] | 0.15 [-0.14, 0.43] |
| Week 24 (follow-up) | (*n* = 66) | (*n* = 64) | 0.45 (1.00) | 0.41 (0.99) | 0.29 [-0.01, 0.60] | 0.35 [0.04, 0.66] |
| **WAIS-IV Coding (Total)** | | | | | | |
| Week 0 (baseline) | (*n* = 73) | (*n* = 74) | 0.06 (1.09) | -0.05 (0.91) | -0.02 [-0.32, 0.27] | -0.05 [-0.34, 0.25] |
| Week 8 (post-intervention) | (*n* = 65) | (*n* = 66) | 0.19 (1.17) | -0.02 (0.88) | 0.06 [-0.23, 0.34] | 0.01 [-0.28, 0.29] |
| Week 24 (follow-up) | (*n* = 67) | (*n* = 64) | 0.32 (1.11) | 0.09 (0.87) | 0.21 [-0.09, 0.52] | 0.12 [-0.19, 0.43] |
| **Category fluency (Total)** | | | | | | |
| Week 0 (baseline) | (*n* = 73) | (*n* = 74) | 0.03 (1.02) | -0.03 (0.98) | 0.01 [-0.33, 0.34] | -0.05 [-0.38, 0.29] |
| Week 8 (post-intervention) | (*n* = 67) | (*n* = 66) | 0.07 (0.97) | -0.03 (0.94) | 0.03 [-0.29, 0.34] | 0.00 [-0.32, 0.32] |
| Week 24 (follow-up) | (*n* = 67) | (*n* = 64) | 0.12 (1.02) | 0.08 (1.02) | 0.06 [-0.29, 0.41] | 0.08 [-0.27, 0.44] |
| **Letter fluency (Total)** | | | | | | |
| Week 0 (baseline) | (*n* = 73) | (*n* = 74) | -0.03 (0.87) | 0.03 (1.12) | -0.09 [-0.40, 0.21] | 0.04 [-0.26, 0.35] |
| Week 8 (post-intervention) | (*n* = 67) | (*n* = 66) | -0.02 (0.84) | 0.09 (1.15) | -0.04 [-0.33, 0.24] | 0.09 [-0.20, 0.38] |
| Week 24 (follow-up) | (*n* = 67) | (*n* = 64) | 0.13 (0.97) | 0.24 (1.08) | 0.06 [-0.26, 0.39] | 0.19 [-0.14, 0.52] |
| **TMT-A (Time)** | | | | | | |
| Week 0 (baseline) | (*n* = 73) | (*n* = 74) | 0.03 (0.95) | -0.03 (1.05) | 0.05 [-0.27, 0.36] | 0.02 [-0.30, 0.34] |
| Week 8 (post-intervention) | (*n* = 67) | (*n* = 66) | 0.15 (0.80) | 0.02 (0.99) | 0.05 [-0.25, 0.35] | 0.02 [-0.28, 0.32] |
| Week 24 (follow-up) | (*n* = 67) | (*n* = 64) | 0.08 (0.95) | -0.03 (1.03) | 0.05 [-0.29, 0.38] | 0.01 [-0.33, 0.35] |
| **TMT-B (Time)** | | | | | | |
| Week 0 (baseline) | (*n* = 73) | (*n* = 74) | 0.01 (1.04) | -0.01 (0.97) | -0.03 [-0.29, 0.23] | -0.02 [-0.28, 0.24] |
| Week 8 (post-intervention) | (*n* = 67) | (*n* = 66) | 0.09 (0.90) | 0.05 (0.75) | -0.03 [-0.26, 0.20] | 0.01 [-0.23, 0.24] |
| Week 24 (follow-up) | (*n* = 67) | (*n* = 64) | 0.02 (1.10) | 0.06 (0.80) | -0.03 [-0.31, 0.25] | 0.06 [-0.23, 0.34] |
| **Stroop naming (Time)** | | | | | | |
| Week 0 (baseline) | (*n* = 71) | (*n* = 73) | -0.01 (0.98) | 0.01 (1.02) | -0.09 [-0.34, 0.17] | 0.01 [-0.24, 0.26] |
| Week 8 (post-intervention) | (*n* = 65) | (*n* = 66) | 0.10 (0.88) | 0.11 (1.00) | -0.07 [-0.32, 0.18] | 0.03 [-0.22, 0.27] |
| Week 24 (follow-up) | (*n* = 65) | (*n* = 63) | 0.10 (0.96) | 0.15 (0.93) | -0.05 [-0.31, 0.22] | 0.05 [-0.21, 0.32] |
| **Stroop incongruent (Time)** | | | | | | |
| Week 0 (baseline) | (*n* = 71) | (*n* = 71) | 0.00 (1.07) | 0.00 (0.93) | -0.07 [-0.34, 0.19] | -0.01 [-0.27, 0.26] |
| Week 8 (post-intervention) | (*n* = 65) | (*n* = 65) | 0.15 (0.83) | 0.17 (0.82) | -0.03 [-0.28, 0.22] | 0.08 [-0.17, 0.33] |
| Week 24 (follow-up) | (*n* = 65) | (*n* = 63) | 0.16 (0.91) | 0.26 (0.83) | 0.05 [-0.22, 0.33] | 0.26 [-0.02, 0.54] |
| **Stroop interference (Time)** | | | | | | |
| Week 0 (baseline) | (*n* = 71) | (*n* = 71) | 0.01 (1.12) | -0.01 (0.87) | -0.03 [-0.31, 0.24] | 0.01 [-0.27, 0.28] |
| Week 8 (post-intervention) | (*n* = 65) | (*n* = 65) | 0.13 (0.83) | 0.15 (0.80) | 0.01 [-0.25, 0.26] | 0.10 [-0.15, 0.35] |
| Week 24 (follow-up) | (*n* = 65) | (*n* = 63) | 0.14 (0.89) | 0.23 (0.79) | 0.09 [-0.20, 0.38] | 0.28 [-0.01, 0.58] |
| **MST (Global)** | | | | | | |
| Week 0 (baseline) | (*n* = 66) | (*n* = 66) | -0.11 (0.93) | 0.11 (1.06) | -0.18 [-0.55, 0.18] | 0.04 [-0.32, 0.41] |
| Week 8 (post-intervention) | (*n* = 64) | (*n* = 64) | 0.24 (1.30) | 0.24 (1.11) | -0.03 [-0.36, 0.30] | 0.16 [-0.17, 0.50] |
| Week 24 (follow-up) | (*n* = 67) | (*n* = 61) | 0.27 (1.28) | 0.41 (1.31) | 0.26 [-0.12, 0.65] | 0.40 [0.00, 0.80] |
| **MST (Recognition)** | | | | | | |
| Week 0 (baseline) | (*n* = 66) | (*n* = 67) | -0.02 (1.00) | 0.02 (1.01) | -0.15 [-0.44, 0.15] | -0.08 [-0.37, 0.22] |
| Week 8 (post-intervention) | (*n* = 65) | (*n* = 64) | -0.05 (0.88) | 0.05 (0.86) | -0.14 [-0.41, 0.12] | -0.08 [-0.35, 0.18] |
| Week 24 (follow-up) | (*n* = 67) | (*n* = 61) | 0.00 (1.07) | -0.02 (0.85) | -0.13 [-0.44, 0.18] | -0.10 [-0.42, 0.23] |

The adjusted means presented in the table are from PP analyses. *N*s indicate the number of participants contributing data for that measure/arm/timepoint. Adjusted values are estimated marginal means calculated for linear-time models via the *R* package *emmeans* v.1.7.0. The adjustment is made by averaging over the levels of categorical covariates (site, sex) and by setting the covariate at the mean for continuous covariates (age, education, state anxiety, depressive symptoms). NB: The CRE was omitted from the model used to estimate the adjusted means. All outcomes are standardized, with higher scores representing better performance. Abbreviations: *CMBAS* Caring Mindfulness-Based Approach for Seniors; *HSMP* Health Self-Management Program; *CI* Confidence interval; *SD* Standard deviation; *PACC5_Abridged_* Abridged Preclinical Alzheimer Cognitive Composite 5; *DRS-2* Mattis Dementia Rating Scale-2; *RAVLT* Rey Auditory Verbal Learning Test; *WAIS-IV* Wechsler Adult Intelligence Scale-IV; *TMT* Trail-Making Test; *MST* Mnemonic Similarities Task; *CRE* Cognitive Retest Effect; *PP* Per-protocol.

### Fitted linear mixed models

**Table S3** Linear mixed models fitted using a linear and factorial time specification, respectively (individual outcomes only – see main paper Table 2 for composite outcomes)

| **Measure** | **LMM coefficients (linear-time specification)** | | **LMM coefficients (** **factorial-time specification)** | |
| --- | --- | --- | --- | --- |
|  | **Parameter** | **Estimate [95% CI]** | **Parameter** | **Estimate [95% CI]** |
| **DRS-2 (Total)** | |  | Post-intervention visit | -0.06 [-0.16, 0.05] |
|  | Time (weeks) | 0.11 [-0.01, 0.22] | Follow-up visit | 0.09 [-0.01, 0.19] |
|  | Time × Arm | -0.00 [-0.11, 0.11] | Post-intervention × Arm | **0.13 [0.01, 0.24]** |
|  | Practice | -0.02 [-0.11, 0.08] | Follow-up × Arm | 0.02 [-0.09, 0.13] |
| **RAVLT (Delayed recall)** | |  | Post-intervention visit | **0.13 [0.07, 0.19]** |
|  | Time (weeks) | **0.09 [0.02, 0.16]**^a^ | Follow-up visit | **0.21 [0.15, 0.27]** |
|  | Time × Arm | -0.05 [-0.11, 0.02] | Post-intervention × Arm | 0.01 [-0.06, 0.08] |
|  | Practice | **0.11 [0.05, 0.17]** | Follow-up × Arm | -0.04 [-0.11, 0.03] |
| **WAIS-IV Coding (Total)** | |  | Post-intervention visit | 0.05 [-0.01, 0.10] |
|  | Time (weeks) | **0.08 [0.02, 0.14]** | Follow-up visit | **0.11 [0.06, 0.16]** |
|  | Time × Arm | -0.02 [-0.08, 0.03] | Post-intervention × Arm | -0.00 [-0.06, 0.05] |
|  | Practice | 0.02 [-0.03, 0.07] | Follow-up × Arm | -0.02 [-0.08, 0.03] |
| **Category fluency (Total)** | |  | Post-intervention visit | 0.01 [-0.07, 0.09] |
|  | Time (weeks) | 0.03 [-0.06, 0.12] | Follow-up visit | 0.03 [-0.05, 0.11] |
|  | Time × Arm | 0.03 [-0.06, 0.11] | Post-intervention × Arm | -0.01 [-0.10, 0.08] |
|  | Practice | -0.01 [-0.09, 0.07] | Follow-up × Arm | 0.02 [-0.06, 0.11] |
| **Letter fluency (Total)** | |  | Post-intervention visit | -0.01 [-0.09, 0.08] |
|  | Time (weeks) | 0.08 [-0.02, 0.17] | Follow-up visit | 0.07 [-0.02, 0.15] |
|  | Time × Arm | -0.00 [-0.09, 0.09] | Post-intervention × Arm | 0.02 [-0.07, 0.12] |
|  | Practice | -0.02 [-0.10, 0.06] | Follow-up × Arm | 0.00 [-0.09, 0.09] |
| **TMT-A (Time)** | |  | Post-intervention visit | 0.04 [-0.05, 0.12] |
|  | Time (weeks) | -0.03 [-0.12, 0.07] | Follow-up visit | 0.01 [-0.08, 0.09] |
|  | Time × Arm | -0.00 [-0.10, 0.09] | Post-intervention × Arm | -0.02 [-0.12, 0.07] |
|  | Practice | 0.04 [-0.04, 0.12] | Follow-up × Arm | -0.01 [-0.10, 0.09] |
| **TMT-B (Time)** | |  | Post-intervention visit | 0.04 [-0.05, 0.13] |
|  | Time (weeks) | -0.03 [-0.13, 0.07] | Follow-up visit | 0.01 [-0.08, 0.10] |
|  | Time × Arm | 0.03 [-0.07, 0.12] | Post-intervention × Arm | -0.01 [-0.11, 0.09] |
|  | Practice | 0.04 [-0.04, 0.13] | Follow-up × Arm | 0.02 [-0.08, 0.12] |
| **Stroop naming (Time)** | |  | Post-intervention visit | 0.03 [-0.02, 0.08] |
|  | Time (weeks) | -0.01 [-0.07, 0.04] | Follow-up visit | 0.02 [-0.03, 0.07] |
|  | Time × Arm | 0.00 [-0.05, 0.05] | Post-intervention × Arm | 0.00 [-0.05, 0.05] |
|  | Practice | 0.04 [-0.01, 0.08] | Follow-up × Arm | 0.00 [-0.05, 0.06] |
| **Stroop incongruent (Time)** | | | Post-intervention visit | 0.07 [-0.01, 0.14] |
|  | Time (weeks) | 0.00 [-0.08, 0.09] | Follow-up visit | 0.07 [-0.00, 0.15] |
|  | Time × Arm | 0.05 [-0.03, 0.14]^b^ | Post-intervention × Arm | 0.03 [-0.05, 0.12] |
|  | Practice | **0.08 [0.00, 0.15]**^c^ | Follow-up × Arm | 0.06 [-0.02, 0.14]^d^ |
| **Stroop interference (Time)** | | | Post-intervention visit | 0.06 [-0.03, 0.15] |
|  | Time (weeks) | 0.01 [-0.10, 0.11] | Follow-up visit | 0.07 [-0.02, 0.16] |
|  | Time × Arm | 0.06 [-0.04, 0.16]^e^ | Post-intervention × Arm | 0.04 [-0.06, 0.14] |
|  | Practice | 0.07 [-0.02, 0.16] | Follow-up × Arm | 0.07 [-0.03, 0.16]^f^ |
| **MST (Global)** | |  | Post-intervention visit | **0.16 [0.07, 0.26]** |
|  | Time (weeks) | 0.08 [-0.03, 0.18] | Follow-up visit | **0.20 [0.11, 0.30]** |
|  | Time × Arm | -0.03 [-0.13, 0.08] | Post-intervention × Arm | -0.05 [-0.16, 0.06] |
|  | Practice | **0.11 [0.02, 0.20]** | Follow-up × Arm | -0.04 [-0.14, 0.07] |
| **MST (Recognition)** | |  | Post-intervention visit | -0.02 [-0.12, 0.09] |
|  | Time (weeks) | 0.01 [-0.11, 0.12] | Follow-up visit | 0.01 [-0.09, 0.11] |
|  | Time × Arm | -0.02 [-0.12, 0.09] | Post-intervention × Arm | 0.03 [-0.08, 0.14] |
|  | Practice | 0.00 [-0.09, 0.10] | Follow-up × Arm | -0.01 [-0.12, 0.10] |

The regression coefficients reported here are standardized. The model fits presented in the table are PP analyses. The time metric for linear-time models was weeks (continuous), and for factorial-time models visits (factor). For factorial-time models, the reference visit is baseline. The post-intervention visit was at week 8, and the follow-up visit was at week 24. For both types of model, the reference trial arm is HSMP; positive coefficients for the interaction terms thus represent a relatively greater improvement in the HSMP (vs. CMBAS) arm; negative coefficients indicate the converse. **Emboldened** coefficient estimates had *p*-values < .05 in initial models. All models were adjusted for sex, age, years of education, state anxiety, depressive symptoms and trial site; models using the linear-time specification were also adjusted for cognitive retest effects. Superscripts: ^a^This coefficient was 0.07 [-0.00, 0.15] in the sensitivity model only including participants who attended ≥ 4 intervention sessions (*p* = .054). ^b^This coefficient was 0.11 [0.03, 0.20] in the sensitivity model (*p* = .01). ^c^This coefficient was 0.05 [-0.02, 0.13] in the sensitivity model (*p* = .15). ^d^This coefficient was 0.12 [0.03, 0.21] in the sensitivity model (*p* < .01). ^e^This coefficient was 0.12 [0.02, 0.23] in the sensitivity model (*p* = .02). ^f^This coefficient was 0.14 [0.03, 0.24] in the sensitivity model (*p* = .01). No other model parameters were substantively altered in sensitivity analyses. Abbreviations: *CMBAS* Caring Mindfulness-Based Approach for Seniors; *HSMP* Health Self-Management Program; *CI* Confidence interval; *DRS-2* Mattis Dementia Rating Scale-2; *RAVLT* Rey Auditory Verbal Learning Test; *WAIS-IV* Wechsler Adult Intelligence Scale-IV; *TMT* Trail-Making Test; *MST* Mnemonic Similarities Task; *LMM* Linear mixed model; *PP* Per-protocol.

### Predicting response to interventions

**Table S4** Association between candidate predictors and baseline (week 0) to follow-up (week 24) change scores on composite outcomes, broken down by trial arm

|  | **β [95% CI]** | | | | | | |
| --- | --- | --- | --- | --- | --- | --- | --- |
| **Predictor/**  **Outcome** | **CMBAS** | | |  | **HSMP** | | |
|  | PACC5_Abridged_ | Attention composite | Executive composite |  | PACC5_Abridged_ | Attention composite | Executive composite |
| Age | -0.06 [-0.31, 0.19] | -0.22 [-0.47, 0.03] | -0.12 [-0.37, 0.13] |  | -0.19 [-0.46, 0.08] | -0.20 [-0.45, 0.05] | 0.07 [-0.20, 0.34] |
| Sex^#^ | **-0.33 [-0.60, -0.06]** | -0.12 [-0.39, 0.15] | -0.11 [-0.38, 0.16] |  | 0.06 [-0.27, 0.39] | 0.00 [-0.31, 0.31] | 0.17 [-0.16, 0.50] |
| Education | -0.10 [-0.35, 0.15] | 0.05 [-0.20, 0.30] | -0.07 [-0.32, 0.18] |  | 0.09 [-0.18, 0.36] | -0.08 [-0.35, 0.19] | -0.07 [-0.36, 0.22] |
| Site^$^ |  |  |  |  |  |  |  |
| Cologne | 0.13 [-0.16, 0.42] | -0.14 [-0.43, 0.15] | -0.27 [-0.56, 0.02] |  | -0.08 [-0.39, 0.23] | 0.28 [-0.01, 0.57] | -0.25 [-0.56, 0.06] |
| London | 0.12 [-0.19, 0.43] | -0.06 [-0.37, 0.25] | 0.00 [-0.31, 0.31] |  | -0.23 [-0.54, 0.08] | 0.18 [-0.11, 0.47] | -0.21 [-0.52, 0.10] |
| Lyon | -0.05 [-0.34, 0.24] | 0.05 [-0.24, 0.34] | -0.04 [-0.33, 0.25] |  | -0.09 [-0.44, 0.26] | **0.42 [0.09, 0.75]** | -0.13 [-0.48, 0.22] |
| GDS-15 | -0.15 [-0.40, 0.10] | 0.06 [-0.21, 0.33] | **-0.37 [-0.62, -0.12]** |  | -0.27 [-0.54, 0.00] | -0.23 [-0.48, 0.02] | -0.07 [-0.36, 0.22] |
| STAI-A | 0.20 [-0.11, 0.51] | 0.30 [-0.01, 0.61] | -0.26 [-0.57, 0.05] |  | 0.05 [-0.22, 0.32] | -0.06 [-0.31, 0.19] | 0.07 [-0.20, 0.34] |
| CEQ-credibility | **0.32 [0.03, 0.61]** | 0.15 [-0.16, 0.46] | 0.24 [-0.07, 0.55] |  | 0.00 [-0.29, 0.29] | 0.05 [-0.22, 0.32] | -0.02 [-0.31, 0.27] |
| CEQ-expectancy | 0.26 [-0.03, 0.55] | 0.28 [-0.01, 0.57] | 0.23 [-0.06, 0.52] |  | -0.14 [-0.41, 0.13] | 0.11 [-0.14, 0.36] | -0.19 [-0.46, 0.08] |
| Baseline score | 0.01 [-0.30, 0.32] | **-0.39 [-0.70, -0.08]** | **-0.61 [-0.88, -0.34]** |  | -0.35 [-0.70, 0.00] | -0.23 [-0.50, 0.04] | **-0.68 [-0.99, -0.37]** |

The model fits presented in the table are PP analyses. The regression coefficients reported here are standardized. All analyses were adjusted for age, sex, education and site. **Emboldened** coefficient estimates had *p*-values < .05. Superscripts: ^#^Reference sex is female. ^$^Reference site is Barcelona. Abbreviations: *PACC5_Abridged_* Abridged Preclinical Alzheimer Cognitive Composite 5; *CMBAS* Caring Mindfulness-Based Approach for Seniors; *HSMP* Health Self-Management Program; *CI* Confidence interval; *β* Standardized linear regression coefficient; *GDS-15* 15-item Geriatric Depression Scale; *STAI-A* State-Trait Anxiety Inventory-State subscale; *CEQ* Credibility/Expectancy Questionnaire; *PP* Per-protocol.

**Figure S2** Estimated change in individual cognitive tests for each trial arm (linear-time specification)


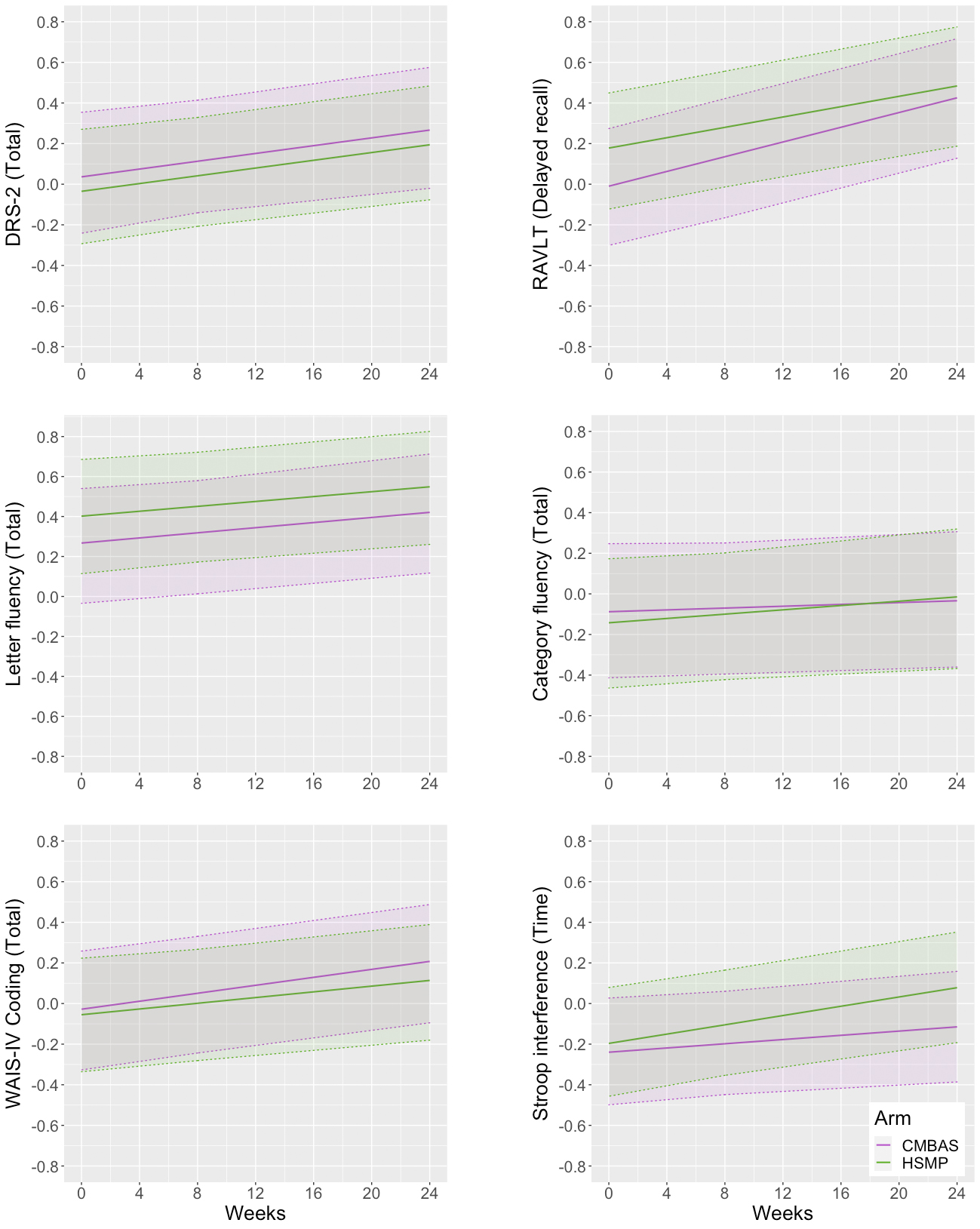


The graphs visualize the trajectories modeled using the PP linear-time LMMs. The cognitive retest effect parameters were omitted from the models graphed, as these resulted in discontinuous trajectories. All outcomes are standardized, with higher scores representing better performance. To render the figures intelligibly, data are for a ‘prototypical’ female participant with sample grand mean values for age, education state anxiety and depressive symptoms, at the Barcelona site. The shaded areas are 95% confidence intervals for the fixed effects. Abbreviations: *DRS-2* Mattis Dementia Rating Scale-2, *RAVLT* Rey Auditory Verbal Learning Test, *WAIS-IV,* Wechsler Adult Intelligence Scale-IV, *CMBAS* Caring Mindfulness-Based Approach for Seniors, *HSMP* Health Self-Management Program; *PP* Per-protocol.

**Figure S3** Estimated change in individual cognitive tests for each trial arm (linear-time specification)


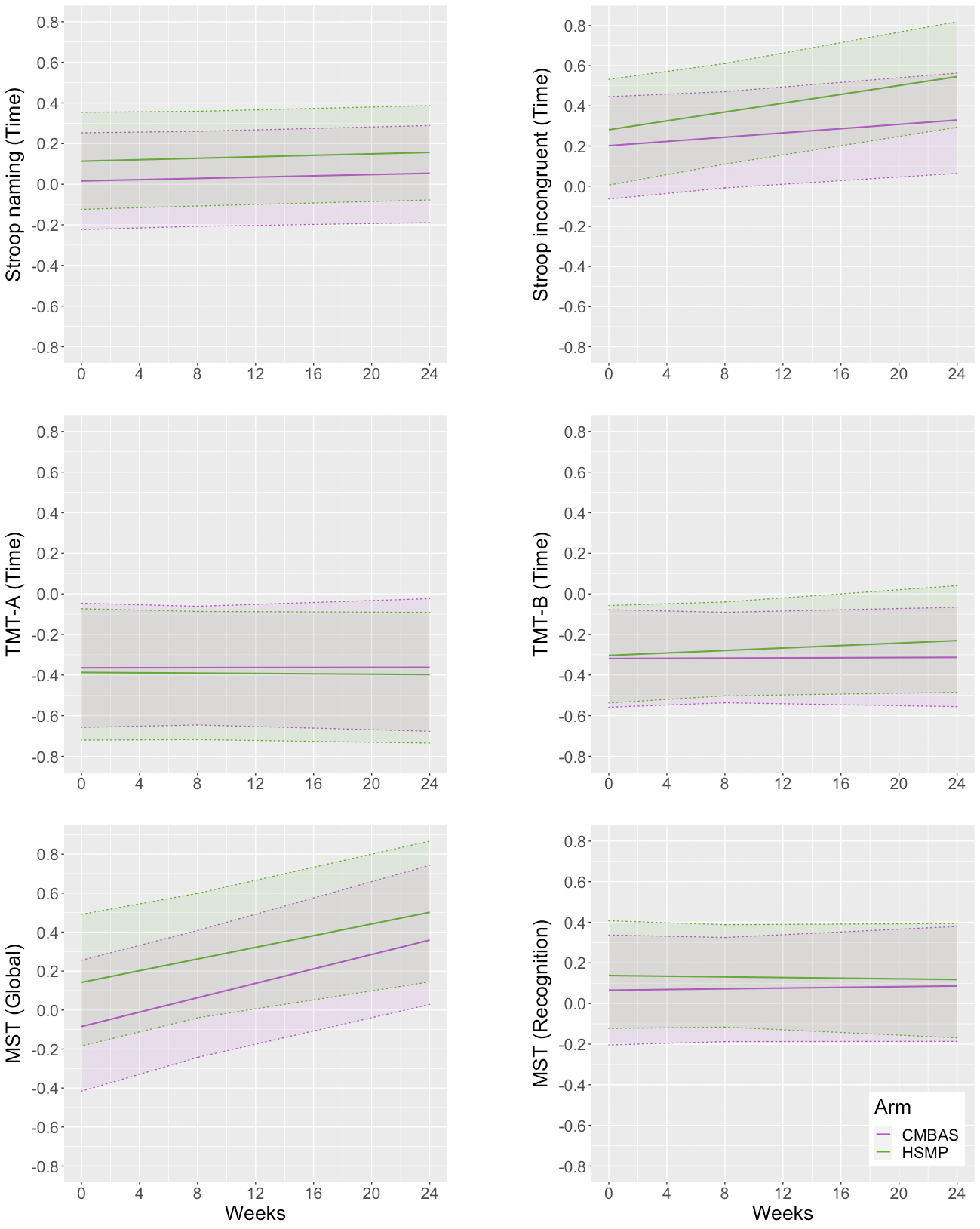


The graphs visualize the trajectories modeled using the PP linear-time LMMs. The cognitive retest effect parameters were omitted from the models graphed, as these resulted in discontinuous trajectories. All outcomes are standardized, with higher scores representing better performance. To render the figures intelligibly, data are for a ‘prototypical’ female participant with sample grand mean values for age, education state anxiety and depressive symptoms, at the Barcelona site. NB: for ‘Stroop naming (Time)’, a different reference site was used (Lyon), as the fixed intercepts for CMBAS and HSMP were too great to be graphed on the same scale as the other outcome figures [CMBAS=0.9; HSMP=1.0]. The shaded areas are 95% confidence intervals for the fixed effects. Abbreviations: *TMT* Trail-Making Test, *MST* Mnemonic Similarities Task, *CMBAS* Caring Mindfulness-Based Approach for Seniors, *HSMP* Health Self-Management Program; *PP* Per-protocol.

## Intention-to-treat analyses

### Fitted linear mixed models

**Table S5** Pooled linear mixed models derived from analyses of multiply-imputed SCD-Well trial data (m = 5)

*Please note – in contrast to the results reported elsewhere, these coefficients are unstandardized (see NB below table)*

| **Measure** | | **LMM coefficients (linear-time specification)** | | **LMM coefficients (** **factorial-time specification)** | |
| --- | --- | --- | --- | --- | --- |
|  |  | **Parameter** | **Estimate [95% CI]** | **Parameter** | **Estimate [95% CI]** |
| **PACC5_Abridged_** | | |  | Post-intervention visit | 0.109 [-0.015, 0.233] |
|  | Time (weeks) | | **0.011 [0.004, 0.017]** | Follow-up visit | **0.325 [0.195, 0.456]** |
|  | Time × Arm | | -0.001 [-0.009, 0.006] | Post-intervention × Arm | 0.108 [-0.068, 0.283] |
|  | Practice | | 0.082 [-0.036, 0.200] | Follow-up × Arm | -0.002 [-0.184, 0.179] |
| **Attention composite** | | |  | Post-intervention visit | **0.136 [0.015, 0.257]** |
|  | Time (weeks) | | 0.003 [-0.003, 0.009] | Follow-up visit | **0.167 [0.043, 0.292]** |
|  | Time × Arm | | -0.002 [-0.009, 0.006] | Post-intervention × Arm | -0.054 [-0.227, 0.118] |
|  | Practice | | 0.091 [-0.021, 0.203] | Follow-up × Arm | -0.046 [-0.221, 0.128] |
| **Executive composite** | | |  | Post-intervention visit | 0.077 [-0.079, 0.233] |
|  | Time (weeks) | | 0.002 [-0.007, 0.010] | Follow-up visit | 0.133 [-0.024, 0.289] |
|  | Time × Arm | | 0.005 [-0.005, 0.014] | Post-intervention × Arm | 0.110 [-0.109, 0.330] |
|  | Practice | | 0.101 [-0.045, 0.247] | Follow-up × Arm | 0.124 [-0.104, 0.353] |
| **DRS-2 (Total)** | | |  | Post-intervention visit | -0.110 [-0.331, 0.110] |
|  | | Time (weeks) | 0.010 [-0.002, 0.022] | Follow-up visit | 0.188 [-0.034, 0.409] |
|  | | Time × Arm | 0.000 [-0.013, 0.013] | Post-intervention × Arm | **0.338 [0.023, 0.653]** |
|  | | Practice | -0.021 [-0.228, 0.185] | Follow-up × Arm | 0.067 [-0.243, 0.376] |
| **RAVLT (Delayed recall)** | | |  | Post-intervention visit | **0.278 [0.142, 0.413]** |
|  | | Time (weeks) | **0.009 [0.002, 0.016]** | Follow-up visit | **0.444 [0.305, 0.582]** |
|  | | Time × Arm | -0.004 [-0.012, 0.004] | Post-intervention × Arm | 0.031 [-0.159, 0.220] |
|  | | Practice | **0.240 [0.116, 0.364]** | Follow-up × Arm | -0.087 [-0.285, 0.111] |
| **WAIS-IV Coding (Total)** | | |  | Post-intervention visit | 0.105 [-0.008, 0.219] |
|  | | Time (weeks) | **0.008 [0.002, 0.014]** | Follow-up visit | **0.226 [0.113, 0.339]** |
|  | | Time × Arm | -0.003 [-0.009, 0.004] | Post-intervention × Arm | -0.029 [-0.202, 0.145] |
|  | | Practice | 0.039 [-0.066, 0.145] | Follow-up × Arm | -0.064 [-0.225, 0.097] |
| **Category fluency (Total)** | | |  | Post-intervention visit | 0.040 [-0.136, 0.216] |
|  | | Time (weeks) | 0.004 [-0.006, 0.013] | Follow-up visit | 0.078 [-0.108, 0.263] |
|  | | Time × Arm | 0.004 [-0.007, 0.015] | Post-intervention × Arm | -0.030 [-0.270, 0.210] |
|  | | Practice | -0.021 [-0.189, 0.147] | Follow-up × Arm | 0.078 [-0.188, 0.343] |
| **Letter fluency (Total)** | | |  | Post-intervention visit | -0.016 [-0.195, 0.163] |
|  | | Time (weeks) | 0.009 [-0.002, 0.019] | Follow-up visit | 0.154 [-0.032, 0.340] |
|  | | Time × Arm | -0.001 [-0.013, 0.011] | Post-intervention × Arm | 0.069 [-0.182, 0.320] |
|  | | Practice | -0.045 [-0.213, 0.124] | Follow-up × Arm | -0.015 [-0.305, 0.276] |
| **TMT-A (Time)** | | |  | Post-intervention visit | 0.111 [-0.081, 0.303] |
|  | | Time (weeks) | -0.001 [-0.011, 0.010] | Follow-up visit | 0.073 [-0.120, 0.267] |
|  | | Time × Arm | -0.002 [-0.013, 0.010] | Post-intervention × Arm | -0.078 [-0.352, 0.197] |
|  | | Practice | 0.084 [-0.100, 0.268] | Follow-up × Arm | -0.049 [-0.331, 0.232] |
| **TMT-B (Time)** | | |  | Post-intervention visit | 0.064 [-0.128, 0.255] |
|  | | Time (weeks) | -0.004 [-0.015, 0.007] | Follow-up visit | -0.013 [-0.215, 0.189] |
|  | | Time × Arm | 0.004 [-0.008, 0.016] | Post-intervention × Arm | 0.015 [-0.259, 0.288] |
|  | | Practice | 0.089 [-0.096, 0.274] | Follow-up × Arm | 0.095 [-0.195, 0.385] |
| **Stroop naming (Time)** | | |  | Post-intervention visit | 0.088 [-0.028, 0.203] |
|  | | Time (weeks) | 0.000 [-0.006, 0.005] | Follow-up visit | 0.075 [-0.040, 0.190] |
|  | | Time × Arm | 0.001 [-0.006, 0.007] | Post-intervention × Arm | -0.015 [-0.172, 0.143] |
|  | | Practice | 0.081 [-0.030, 0.192] | Follow-up × Arm | 0.010 [-0.147, 0.167] |
| **Stroop incongruent (Time)** | | | | Post-intervention visit | 0.130 [-0.028, 0.287) |
|  | | Time (weeks) | -0.001 [-0.010, 0.007] | Follow-up visit | 0.136 [-0.024, 0.295] |
|  | | Time × Arm | 0.005 [-0.004, 0.014] | Post-intervention × Arm | 0.104 [-0.119, 0.326] |
|  | | Practice | **0.169 [0.023, 0.315]** | Follow-up × Arm | 0.142 [-0.080, 0.364] |
| **Stroop interference (Time)** | | | | Post-intervention visit | 0.102 [-0.086, 0.290) |
|  | | Time (weeks) | -0.001 [-0.012, 0.009] | Follow-up visit | 0.117 [-0.077, 0.311] |
|  | | Time × Arm | 0.006 [-0.005, 0.017] | Post-intervention × Arm | 0.130 [-0.142, 0.402] |
|  | | Practice | 0.152 [-0.026, 0.331] | Follow-up × Arm | 0.161 [-0.103, 0.425] |
| **MST (Global)** | | |  | Post-intervention visit | **0.280 [0.081, 0.479]** |
|  | | Time (weeks) | 0.010 [-0.002, 0.022] | Follow-up visit | **0.402 [0.191, 0.614]** |
|  | | Time × Arm | -0.002 [-0.015, 0.010] | Post-intervention × Arm | -0.118 [-0.416, 0.179] |
|  | | Practice | 0.150 [-0.043, 0.343] | Follow-up × Arm | -0.079 [-0.388, 0.230] |
| **MST (Recognition)** | | |  | Post-intervention visit | -0.007 [-0.208, 0.194] |
|  | | Time (weeks) | -0.003 [-0.014, 0.009] | Follow-up visit | -0.014 [-0.233, 0.205] |
|  | | Time × Arm | 0.000 [-0.014, 0.014] | Post-intervention × Arm | 0.092 [-0.198, 0.381] |
|  | | Practice | 0.059 [-0.128, 0.247] | Follow-up × Arm | 0.024 (-0.313, 0.361] |

# ­­

**NB:** In contrast to the other regression models presented in tabular format, the above coefficients are **unstandardized** – it was not possible to extract standardized regression coefficients, because this *R* function is currently unavailable for pooled linear mixed models estimated on multiply-imputed data. All models included the full sample of 147 participants with SCD. The time metric for linear-time models was weeks (continuous), and for factorial-time models visits (factor). For factorial-time models, the reference visit is baseline. The post-intervention visit was at week 8, and the follow-up visit was at week 24. For both types of model, the reference trial arm is HSMP; positive coefficients for the interaction terms thus represent a relatively greater improvement in the HSMP (vs. CMBAS) arm; negative coefficients indicate the converse. **Emboldened** coefficient estimates had *p*-values < .05. All models were adjusted for sex, age, years of education, state anxiety, depressive symptoms and trial site; models using the linear-time specification were also adjusted for cognitive retest effects. Abbreviations: *CMBAS* Caring Mindfulness-Based Approach for Seniors; *HSMP* Health Self-Management Program; *CI* Confidence interval; *DRS-2* Mattis Dementia Rating Scale-2; *RAVLT* Rey Auditory Verbal Learning Test; *WAIS-IV* Wechsler Adult Intelligence Scale-IV; *TMT* Trail-Making Test; *MST* Mnemonic Similarities Task; *LMM* Linear mixed model; *m* number of imputed datasets; *SCD* Subjective Cognitive Decline.

# References

1. Jurica SJ, Leitten CL, Mattis S: *Dementia Rating Scale-2: Professional manual.* Odessa, FL: Psychological Assessment Resources; 2001.

2. Strauss E, Sherman EMS, Spreen O: *A compendium of neuropsychological tests: administration, norms, and commentary.* 3rd ed. edn. New York; Oxford: Oxford University Press; 2006.

3. Jaeger J: Digit Symbol Substitution Test: The Case for Sensitivity Over Specificity in Neuropsychological Testing. *J Clin Psychopharmacol* 2018, 38:513-519.

4. Wechsler D: *Manual for the Wechsler Adult Intelligence Scale – (4th ed.).* San Antonio, TX: Pearson; 2008.

5. Shao Z, Janse E, Visser K, Meyer AS: What do verbal fluency tasks measure? Predictors of verbal fluency performance in older adults. *Frontiers in Psychology* 2014, 5.

6. Godefroy O, GREFEX: *Fonctions exécutives et pathologies neurologiques et psychiatriques: Évaluation en pratique clinique.* Marseille: Solal; 2008.

7. Scarpina F, Tagini S: The Stroop Color and Word Test. *Frontiers in Psychology* 2017, 8.

8. Stark SM, Yassa MA, Lacy JW, Stark CE: A task to assess behavioral pattern separation (BPS) in humans: Data from healthy aging and mild cognitive impairment. *Neuropsychologia* 2013, 51:2442-2449.

9. Vivot A, Power MC, Glymour MM, Mayeda ER, Benitez A, Spiro A, 3rd, Manly JJ, Proust-Lima C, Dufouil C, Gross AL: Jump, hop, or skip: modeling practice effects in studies of determinants of cognitive change in older adults. *Am J Epidemiol* 2016, 183:302-314.

10. Diagnosing collinearity in mixed models from lme4 [<https://hlplab.wordpress.com/2011/02/24/diagnosing-collinearity-in-lme4/>]

11. Buuren Sv: Chapter 11: Longitudinal data. In *Flexible Imputation of Missing Data, Second Edition.* Boca Raton, FL: CRC Press; 2018

12. Austin PC, White IR, Lee DS, van Buuren S: Missing Data in Clinical Research: A Tutorial on Multiple Imputation. *Can J Cardiol* 2021, 37:1322-1331.

13. Kumle L, Vo ML, Draschkow D: Estimating power in (generalized) linear mixed models: An open introduction and tutorial in R. *Behav Res Methods* 2021, 53:2528-2543.

14. Scenario 3: Having strong and detailed a priori assumptions [<https://lkumle.github.io/power_notebooks/Scenario3_notebook.html>]

15. Plate JDJ, Borggreve AS, van Hillegersberg R, Peelen LM: Post Hoc Power Calculation: Observing the Expected. *Ann Surg* 2019, 269:e11.

16. Heckman MG, Davis JM, 3rd, Crowson CS: Post Hoc Power Calculations: An Inappropriate Method for Interpreting the Findings of a Research Study. *J Rheumatol* 2022.

17. Zhang Y, Hedo R, Rivera A, Rull R, Richardson S, Tu XM: Post hoc power analysis: is it an informative and meaningful analysis? *Gen Psychiatr* 2019, 32:e100069.

18. Wolfsgruber S, Kleineidam L, Guski J, Polcher A, Frommann I, Roeske S, Spruth EJ, Franke C, Priller J, Kilimann I, et al: Minor neuropsychological deficits in patients with subjective cognitive decline. *Neurology* 2020, 95:e1134-e1143.

19. Marchant NL, Barnhofer T, Coueron R, Wirth M, Lutz A, Arenaza-Urquijo EM, Collette F, Poisnel G, Demnitz-King H, Schild AK, et al: Effects of a mindfulness-based intervention versus health self-management on subclinical anxiety in older adults with subjective cognitive decline: the SCD-Well randomized superiority trial. *Psychother Psychosom* 2021, 90:341-350.

20. Mindfulness intervention for mild cognitive impairment led to attention-related improvements and neuroplastic changes: results from a 9-month randomized control trial [Internet] [Available from: osf.io/ykz2c]
